# Recent Advances in Nanocellulose Composites with Polymers: A Guide for Choosing Partners and How to Incorporate Them

**DOI:** 10.3390/polym10050517

**Published:** 2018-05-10

**Authors:** Arindam Chakrabarty, Yoshikuni Teramoto

**Affiliations:** 1 Department of Applied Biological Sciences, Gifu University, Gifu 501-1193, Japan; arindam.polym@gmail.com; 2 Center for Highly Advanced Integration of Nano and Life Sciences (G-CHAIN), Gifu University, Gifu 501-1193, Japan

**Keywords:** cellulose nanocrystal, cellulose nanofiber, polymer nanocomposite, polymer–filler interaction

## Abstract

In recent years, the research on nanocellulose composites with polymers has made significant contributions to the development of functional and sustainable materials. This review outlines the chemistry of the interaction between the nanocellulose and the polymer matrix, along with the extent of the reinforcement in their nanocomposites. In order to fabricate well-defined nanocomposites, the type of nanomaterial and the selection of the polymer matrix are always crucial from the viewpoint of polymer–filler compatibility for the desired reinforcement and specific application. In this review, recent articles on polymer/nanocellulose composites were taken into account to provide a clear understanding on how to use the surface functionalities of nanocellulose and to choose the polymer matrix in order to produce the nanocomposite. Here, we considered cellulose nanocrystal (CNC) and cellulose nanofiber (CNF) as the nanocellulosic materials. A brief discussion on their synthesis and properties was also incorporated. This review, overall, is a guide to help in designing polymer/nanocellulose composites through the utilization of nanocellulose properties and the selection of functional polymers, paving the way to specific polymer–filler interaction.

## 1. Introduction

The development of high-performance and/or functional polymer materials using nanoparticles or nanofillers has already become a prominent area of current research not only in academia, but also in industry. In this decade, the selection of the nanofiller for preparing nanocomposites has raised concerns with respect to the environment. There is an immense need felt to introduce sustainable and biodegradable nanofillers. In this regard, cellulosic nanomaterials have attained a great deal of research interest because of their natural abundance and biodegradability, as well as many other important inherent features that lead to functionality expression and performance enhancement as materials.

The preparation of polymer nanocomposites using nanocelluloses has been found to be of growing interest due to the unique characteristics of those nanomaterials, such as numerous surface –OH groups and their associated ease of surface modification, high strength, (potentially) low cost, and renewability. However, those nanomaterials also suffer from certain disadvantages, including high moisture adsorption and poor compatibility with the hydrophobic polymer matrix. It has, thus, been necessitated to introduce any sort of interaction, either covalent or non-covalent, between the polymer and nanocellulose in order to achieve the desired performance and functionalities. The type and extent of the interaction are the prime factors for the dispersibility of the nanocellulose in the polymer matrix and, subsequently, for the final properties of the nanocomposite. 

The original specialized fields of the researchers who have contributed to the nano-cellulose area are broad: physical chemistry such as colloids and interfaces, mechanical engineering, wood science, plant biology, etc. We can find review articles from the past several years providing an overview on the structure and properties of the cellulosic nanomaterials [1] and their surface modification [2,3,4]. In terms of the properties of nanocellulose, their colloidal behaviour has also been focused upon [5,6]. For the practical use of nanocelluloses, meanwhile, the route of their compounding with synthetic polymers is realistic. Prior to nanocellulosic polymer composites, studies on the molecular composites of cellulose and its derivatives with polymers had made significant progress since the late 1980s [7,8]. It should be pointed out that those findings are highly suggestive for the researchers trying to enter the field of nanocellulose. There are quite a few reviews summarizing the advances in polymer nanocomposites reinforced by nanocellulose, as well. Those reviews place the entire emphasis on the preparation and properties of cellulosic bio-nanocomposites [9], achieved mechanical properties [10], the comparison of mechanical reinforcement with the type and content of nanocellulose [11], and the processing of nanocellulosic composites [12,13,14]. However, the reviews did not appear to highlight the nature of polymer-nanocellulose interactions that resulted in the desired reinforcement.

In this context, the authors, as a group familiar with cellulose science and polymer synthesis, are particularly interested in exploring the types of interactions present in the reported polymer/nanocellulose composites. Considering the recent literature on polymer/nanocellulose composites, the purpose of this review is to determine the possible modes of interaction, such as covalent or non-covalent interactions between the polymer and nanocellulose, and to classify those examples accordingly. In some cases, the polymer-grafted nanocellulose is important for the development of polymer/nanocellulose composites [15,16]. Since the sort of interaction has a direct influence over the final properties of the nanocomposite, this review will also give a clear understanding of the polymer–filler interaction and the properties of the prepared nanocomposites, including the specific applications. Nonetheless, if the authors can systematize how to incorporate them, possible performances and functionalities expand by choosing adequate constituents (monomers); this might sound obvious, though. In this review, the authors would like to provide suggestions regarding where the next ideas will emerge.

## 2. Nanocellulose: Availability and Properties

Cellulose is a natural high molecular weight homopolymer which is composed of β 1–4 linked anhydro D-glucose units. Considering the direction perpendicular to the molecular chain, cellulose does not naturally exist as one molecule, but crystallizes via intermolecular hydrogen bonding to form microfibrils. The natural crystal system is cellulose I [3]. Highly-crystalline nanocelluloses are obtained as a minimum unit by defibrillation and/or chemical treatment. As long as extreme destruction of the chemical structure is not involved, the nanocelluloses cannot be loosened any more, in principle. Since the end of the last century, nanocelluloses have been attracting attention as efficient methods were re-evaluated to be extracted as, or from, the microfibrils. 

From the viewpoint of biosynthesis, the unit of a bundle of cellulose molecular chains constituting microfibrils is determined by the arrangement of cellulose synthase in the cell membrane. The polymerized cellulose is extruded from the terminal enzyme complexes (TC), where the number of cellulose chains included in a microfibril is the product of the numbers of synthetic sites constituting TC and synthetic proteins at each site [1,17]. The number and arrangement of the synthetic sites vary depending on species. In the case of wood, the theory that 6 × 6 cellulose molecular chains are bundled has been accepted, whereas there are recent reports that higher plants synthesize cellulose microfibrils that initially comprise 18 chains [18]. Since the series of reports is very recent, the authors here avoid getting into detail, but the discussion will further deepen in the future.

When viewed in the fiber direction, in higher plants, crystalline regions and regions without long-range order (amorphous) appear along the microfibrils. This periodic structure is related to what has been known for a long time as the level-off degree of polymerization (LODP) [19]. LODP manifests as highly crystalline particles with 200–300 residues produced by pulp beating or enzymatic or acid treatment. Various concepts on the position of the amorphous part have been proposed, but the studies to clarify the exact structure are continuing.

The family of cellulosic nanomaterials consists of cellulose nanocrystal (CNC) collected by controlled acidic hydrolysis, cellulose nanofiber (CNF) obtained by chemical treatment and mechanical disintegration, and bacterial cellulose produced by *Acetobacter xylinum* [20]. Their ultimate dimensions and properties always depend on the type of cellulosic source and the extraction conditions [20]. In this review, the authors deal with CNC and CNF, which are promising industrial raw materials with news on the market entries. Incidentally, CNC is supplied by several research companies [21], while CNF manufacturing and sale are done by paper, machine, and chemical companies [22].

Aggregation is a common problem in using CNC and CNF [1,2,3,4,5,6,9,10,11,12,13,14,20]. The authors have already mentioned that cellulose molecules tend to aggregate through hydrogen bonds, which lead to crystallization, but the ease of aggregation is remarkable even on the scale of nanocellulose. In order to avoid aggregation, the nanocellulose, generally supplied as an aqueous dispersion, should not be inadvertently dried. For combining with polymers, it is ideal to subject nanocelluloses to a process in a never-dry state. However, lyophilized ones are often used. Even if nanocelluloses are freeze-dried, there is a concern of aggregation. However, if water in the aqueous dispersion is substituted with *tert*-butanol and lyophilized, agglomeration is suppressed to a certain extent.

### 2.1. Cellulose Nanocrystal (CNC)

From the 1940s to the 1950s, Nickerson and Habrle first observed the degradation of cellulosic fibers in boiling acidic solutions [23] and, subsequently, Ranby performed the controlled sulfuric acid hydrolysis of cellulosic fibers, finally obtaining the colloidal suspension of cellulosic crystals [24,25]. The transmission electron microscopic (TEM) observation of the hydrolyzed suspension revealed the formation of needle-like particles. Further X-ray diffraction analysis showed a similar crystalline structure as pristine cellulose [26]. This crystalline rigid and needle-like cellulosic segment is now called cellulose nanocrystal (CNC), or whiskers. Then, in 1959, Marchessault et al. discovered the birefringence of the aqueous dispersion above the critical concentration, and in the 1990s Revol et al. systematically reported chiral nematic liquid crystallinity. In the 2000s, research on CNC as a filler of polymer composite materials became active.

**Scheme 1:**
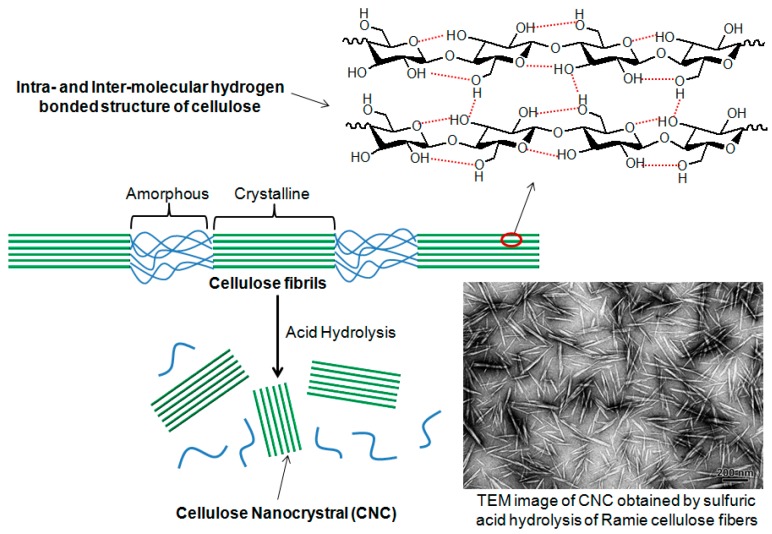
Preparation of CNC by acid hydrolysis and the corresponding TEM image (the TEM image is reproduced with the permission from [39]).

As mentioned above, cellulose microfibrils consist of alternating amorphous and crystalline regions. The amorphous regions are considered as the structural defects or disordered regions in cellulose. Those defects can be preferentially removed by enzymatic [27,28] and acidic hydrolysis [29,30,31]. The amorphous regions undergo faster hydrolysis compared to the crystalline segments [32]. The idea of controlled hydrolysis was, thus, employed for the disruption of the amorphous regions leaving the crystalline part intact (Scheme 1). As a result, highly-crystalline CNC can be obtained.

The preparation of CNC is generally dependant on the type and concentration of the strong acid, the reaction temperature, and time. After the reaction, the resulting suspension is generally diluted with water and washed with repeated centrifugation, followed by dialysis against distilled water to remove the free acid molecules. Sulfuric (64–65%, *w*/*w*) [30] and hydrochloric acids [33] are generally used to perform the hydrolysis reaction. In addition, phosphoric [34] and hydrobromic [35] acids were also found to be reported. In terms of the stability of the aqueous suspension, the CNC obtained by sulfuric acid hydrolysis is better than the one obtained using hydrochloric acid. In the presence of sulfuric acid, the surface –OH groups of CNC partially react with the acid creating charged sulfate ester groups on its surface. The presence of a surface charge is responsible for providing good dispersibility in aqueous suspension. It was seen that CNC can be obtained by the acidic hydrolysis of cellulose fibers derived from several sources, such as cotton [29], tunicate [36], microcrystalline cellulose [37], sisal [38], ramie [39], softwood pulp [40], sugar beet pulp [41], bacterial cellulose [42], chardonnay grape skins [43], rice straw [44], and soy hulls [45].

The physical dimension of CNC, designated by length (*L*), diameter (*D*), and aspect ratio (*L*/*D*), is greatly dependent to the cellulosic source and preparation condition. Generally, CNCs obtained from cotton are shorter with an aspect ratio within 10–30, whereas it is around 70 for those obtained from tunicates. The dimensions of CNCs obtained from wood, cotton, and tunicates are *L* = 100–200 nm/*D* = 3–5 nm, *L* = 100–300 nm/*D* = 5–10 nm, and *L* = 500–2000 nm/*D* = 10–20 nm, respectively [46]. It was observed that the increase in hydrolysis time decreased the length of CNC along with the polydispersity [30]. Similarly, shorter CNCs are obtained with the increase in temperature of the hydrolysis reaction [47]. However, in relation to the above-mentioned LODP, in higher plants it does not reach below a certain length (cellulose from animal-type tunicates does not have LODP).

CNCs possess a large surface area of 250–500 m^2^/g and plenty of surface hydroxyl groups. In addition, they also have a high tensile strength of 7500 MPa and a Young’s modulus of 100–140 GPa [48]. Such properties of CNCs make them suitable reinforcing nanofillers for polymer nanocomposites. Moreover, the surface hydroxyl groups provide the opportunity for the surface modification by small molecules and polymers. Surface modifications by the grafting to, or from, the surface of CNCs not only improve their compatibility in a polymer matrix, but also impart special functionalities for their application in the biomedical, water treatment, electronics, and energy fields [49], as well as other applications.

### 2.2. Cellulose Nanofiber (CNF)

CNF, called micro (nano) fibrillated cellulose as well, is a substance based on cellulose microfibrils as an elementary unit. Although there are numerical variations in the literature [1,2,3,4,5,6,9,10,11,12,13,14,20], it is safe to define CNF as a fibrous material with a width of 4–20 nm, *L*/*D* > 100. CNF generally does not positively contain non-cellulose components. Producing CNF from cell walls of higher plants means peeling off the microfibrils. Production procedures generally include purification and fibrillation of plant raw materials.

Purification of the plant raw materials is carried out for the removal of non-cellulose components; they include pectin, hemicellulose, and lignin as cell wall components. While hemicellulose does not significantly inhibit fibrillation, it is surely inhibited by the large amount of lignin remaining. For woody raw materials, a process of chemical pulping through cooking and bleaching processes is common. In addition, delignification treatment (Wise method, peracetic acid treatment) and hemicellulose extraction with alkali are carried out.

The chemical pulp manufacturing process includes drying and dehydration processes. These inhibit fibrillation. The inhibition is explained by a phenomenon called hornification. Hornification is an irreversible in-fiber aggregation that occurs when pulp fibers are dried or dehydrated. The extent of hornification is generally confirmed by a decrease in the water retention rate when the pulp fibers are re-swollen with water.

Fibrillation is generally performed by treating an aqueous suspension of cellulosic raw materials with various apparatuses. The mechanical energy on the raw material should be spent as much as possible on the peeling of the microfibrils. The energy given to the microfibrils themselves leads to the destruction and breakage thereof. It is desired to obtain a CNF having as narrow distribution of fiber widths as possible without lowering the crystallinity and degree of polymerization of the cellulose.

**Scheme 2:**
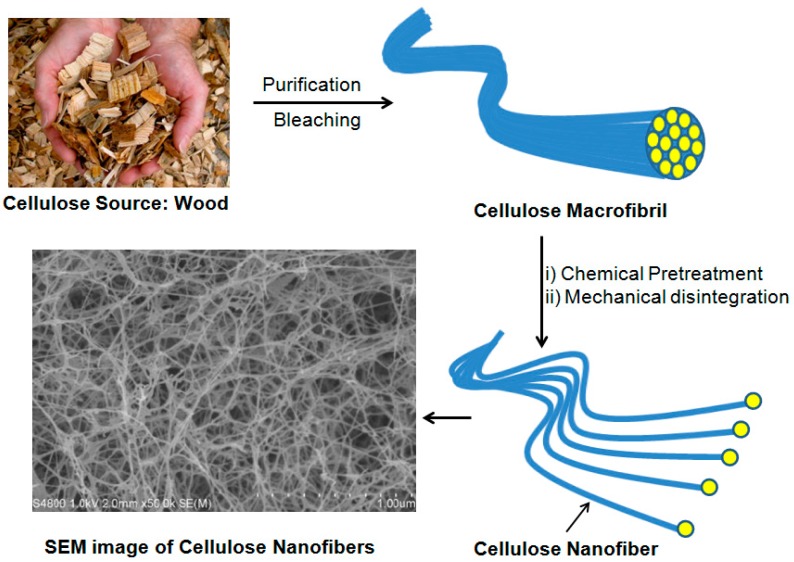
Production of cellulose nanofibers by the combination of chemical and mechanical treatment; the SEM image shows the highly-entangled nanofiber network (the SEM image is reproduced with permission from [65].

CNF production by mechanical fibrillation was reported for the first time for softwood pulp using a high-pressure homogenizer in 1983 [50,51]. Thereafter, a microfluidizer [52] and aqueous counter collision [53] were proposed as methods of pressurizing. Basically, those are chemical-free treatments using water only, and break the intermolecular interaction without destroying the molecular structure. As a result, the nanofibers can be peeled off from the raw materials. A grinder that does not require high pressure is also useful [54,55]. This is a process of passing an aqueous dispersion of cellulosics between two rotating grinding wheels. The grinder to use is also called a wet disk milling apparatus. Frequently-used equipment was originally for grinding for food processing; for example, traditional Japanese “matcha” and “tofu” are made from green tea and soybeans, respectively, by pulverizing using the grinder. Other mechanical fibrillation processes include cryo-crushing [56] and high-speed blending [57] for the production of CNF. In this regard, the recent reviews on CNF production summarizing the mechanical processes are worth citing [58,59]. Scheme 2 shows the general procedure for CNF preparation.

A disadvantage of mechanical fibrillation is that the aqueous dispersion of cellulosic raw material must be treated repeatedly. There is a problem of not only labor and energy, but also the decrease in the degrees of crystallinity and polymerization of cellulose. As a pretreatment to facilitate the fibrillation, mechanical, enzymatic, and chemical treatments are conducted. A refiner is used for mechanical pretreatment [60]. It is interpreted that the primary cell wall is cut by the refiner treatment, which promotes fibrillation for the CNF production. Enzymatic hydrolysis also has the effect of loosening the aggregation of cellulose in the original pulp fiber [61]. As a chemical pretreatment, surface carboxylation by 2,2,6,6-tetramethylpiperidine-1-oxyl radical (TEMPO)-mediated oxidation is very famous [62]. Electrostatic repulsion occurs between negatively-charged microfibrils by selectively converting the primary hydroxyl group at the C6 position on the microfibril surface to a carboxy group. As a result, a homogeneous nanofiber suspension is obtained by a light mechanical treatment. The produced CNF is termed as TEMPO-oxidized CNF, or simply TOCN. The width of the CNF obtained only by the above mechanical treatments is generally ~20 nm or more, but the width of TOCN is 3–5 nm. Such a difference in the widths is an interesting subject related to the cellulose biosynthesis and the stability of nanocelluloses. Other chemical treatments include phosphate esterification [63], carboxymethylation [64], esterification with maleic anhydride (MA) [65], and quaternization [66].

Once the CNF is dried, it is difficult to re-disperse it in water. However, there are examples for preventing aggregation; redispersion of the absolutely-dried CNF by surface carboxymethylation [67], redispersion of the lyophilized product by salt addition [68], and the suppression of drying agglomeration for CNF from fruit peels by leaving pectin contained in the original sample [69].

Similar to CNCs, CNFs can also be obtained from several cellulosic sources [70] that include wood [57], tunicates [71], banana [72], pineapple leaf [73], bamboo [74], cotton [75], algae [76], sludge [77], other industrial residues [78], etc. Their dimensions are very much dependant to the cellulosic source.

The one prepared by electrospinning the solution of cellulose (and its derivatives) is different from the CNF which has been described so far. However, it is sometimes called nanofiber, and it may be encountered in literature searches, etc. The cellulose regenerated from the solution generally has a cellulose II crystal structure rather than the natural cellulose I. Although accuracy should be taken for the word meaning, the authors will cover some of them in this review as well, because it is often possible to deal with them similarly from the viewpoint of polymer composite fabrication. 

## 3. Polymer Nanocomposites Reinforced by CNC

CNCs have attracted considerable attention of polymer researchers for their application as reinforcing fillers in nanocomposites because of many important properties, like biocompatibility, biodegradability, being environmentally benign, natural abundance, light weight, potential for chemical modification, and excellent mechanical strength [46,79,80]. However, they also suffer from a severe drawback of substantial hydrophilicity. As a result, CNCs have very poor compatibility with hydrophobic solvents and hydrophobic polymer matrices [81,82]. In a polymer nanocomposite it is always necessary to have good dispersion of the nanofiller in the polymer matrix to exhibit improved performance compared to the pristine polymer. Thus, the preparation of a nanocomposite using CNC is quite challenging due to its extensive aggregation arising from the lack of compatibility with the polymer. In order to circumvent this problem, it is necessary to introduce any sort of interaction between the polymer and CNCs. So far, there have been several reports on the preparation of polymer/CNC nanocomposites via different approaches to achieve the polymer–filler interaction. In the forthcoming discussion, the authors explain those approaches in two categories viz. covalent and non-covalent interactions. 

### 3.1. Polymer/CNC Nanocomposites with Covalent Interaction

In this topic, the authors discuss the fabrication of different polymer/CNC composites where there is covalent bonding between the polymer and CNC. The covalent bonding between the CNC and polymer refers to the direct attachment of polymer chains onto the CNC surface. In the literature, the authors can find several approaches to covalently attach the polymer to the CNC. The approaches can be categorized as ‘grafting to’ and ‘grafting from’ in terms of the polymer chain. The first approach is associated with the attachment of a prepared polymer through a reactive functionality present in the polymer chain. In this case, during the attachment process, the reacting polymer chains feel considerable steric hindrance to access the reaction site on CNC because of the presence of already-attached polymer chains. As a result, the ‘grafting to’ approach is unable to produce very high graft densities. On the other hand, the ‘grafting from’ approach involves the growth of polymer chains on the surface of the CNC. In this case, generally certain polymerization-initiating species are attached on the CNC surface. In the presence of monomer only, there is a possibility of surface-initiated polymerization to take place producing a well-defined nanocomposite with very high graft density [39]. 

In the cases of employing nonaqueous reaction systems and/or hydrophobic reaction reagents, not only freeze-dried CNC is used as a raw material, but also the dispersion medium of never-dried CNC is often replaced with organic solvents from water. When it is desired to finally carry out the reaction in a hydrophobic organic solvent, the initial dispersion medium, water, is often first exchanged with water-soluble acetone and the solvent exchange is conducted in multiple steps. 

In the forthcoming discussion, the authors have categorized according to different coupling reactions adopted for the polymer-CNC covalent attachment. Herein, the authors discuss some recent approaches and their important characteristics along with the extent of reinforcement. 

#### 3.1.1. Urethanization

Isocyanate (–NCO) functionalities are highly reactive to –OH groups producing a urethane linkage (–COONH). This chemistry was successfully utilized to fabricate polymer/CNC nanocomposites having urethane linkages between the polymer and CNC. An early approach was made by Habibi and Dufresne, who utilized the isocyanate-hydroxyl reaction to graft poly(ɛ-caprolactone) (PCL) onto the CNC surface [83]. In this case, the authors prepared isocyanate-terminated PCL (NCO-PCL) which was subjected to react with the –OH groups present on the CNC surface. The grafting reaction was performed in toluene and was catalyzed by triethylamine (TEA). This was how the surface modification of CNC was done by using PCL via the ‘grafting to’ approach. This PCL-modified CNC was further used as nanofiller to prepare the PCL-CNC composite via the solution mixing process. In the presence of the modified CNC, the nanocomposite exhibited a higher modulus and ductility compared to the unmodified CNC-based nanocomposite. A similar approach was reported for the preparation of PCL-PEG-CNC nanocomposite gel [84]. In this case, both the PCL and PEG were functionalized with terminal –NCO units that further react with CNC to produce the crosslinked nanocomposite. The nanocomposite not only had improved mechanical properties, but also exhibited thermal- and water-induced shape memory properties. 

**Scheme 3:**
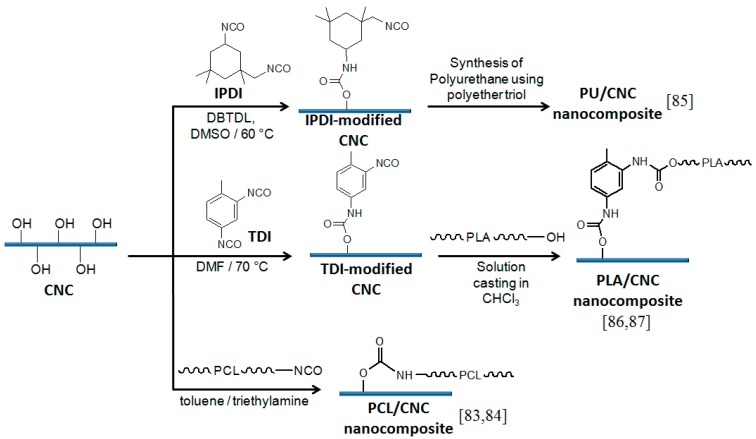
Examples of the reaction between –OH groups of CNC and different –NCO functionalities to produce polymer/CNC nanocomposites.

Girouard et al. adopted a different approach to prepare polyurethane (PU)/CNC nanocomposite where the CNCs were first modified by isophorone diisocyante (IPDI) monomer having two (primary and secondary) –NCO groups with different reactivity to the surface –OH groups of the CNC in the presence of DBTDL catalyst (Scheme 3) [85]. In this case, the secondary –NCO group reacted with the primary –OH group of the CNC, making this an example of site-selective modification of CNC. The successful surface modification of the CNC was confirmed by ATR-FTIR spectroscopy, where there was considerable decrease in –OH peak intensity along with the appearance of the peak for –NCO at 2240 cm^−1^. The unreacted primary –NCO units present on the modified CNC took part in the polymerization in the presence of polyether-based triols to produce PU. The obtained PU-CNC composite showed an increase of about 163% in tensile strength compared to the pristine PU. 

The urethanization reaction was also used to improve the dispersibility of CNC in a polylactic acid (PLA) matrix [86,87]. In this case, the surface modification of the CNC by toluene diisocyanate (TDI) was performed using a DMF dispersion of CNC at 70 °C. The successful CNC modification was confirmed by solid state ^13^C NMR and FT-IR (peak at 2272 cm^−1^ for –NCO) analyses. The modified CNC was used for the preparation of nanocomposites with PLA by solution casting from chloroform. It was expected that the –OH end group of PLA might react with the –NCO of the modified CNC producing urethane linkages between the PLA and CNC, as evident from the increase in the intensity of the FT-IR absorption band of urethane >C=O. As a result, the PLA/CNC nanocomposite not only exhibited higher crystallinity, but also had improved thermal and mechanical properties. The formation of urethane linkages with CNC is also expected for the reinforcement of the PLA/PU blend [88]. In this case, PU was synthesized in the presence of PLA and CNC, where the –OH groups of CNC along with the polyol reacted with the isocyanates used for PU synthesis. Finally, it formed a semi-interpenetrating polymer network (S–IPN) of PU/PLA/CNC nanocomposite, having a 264% increment in storage modulus at 80 °C. Scheme 3 summarizes the examples of urethanization reactions performed for the preparation of polymer/CNC nanocomposites.

#### 3.1.2. Etherification

**Scheme 4:**
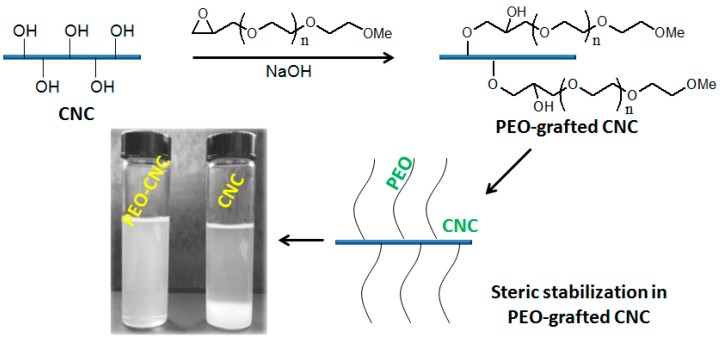
Preparation of PEO-grafted CNC via the etherification reaction, with the improvement in aqueous dispersibility of CNC through steric stabilization (the photograph of CNC dispersion is reproduced with the permission from [90].

Epoxides are very much susceptible to nucleophilic addition. It was found that surface –OH groups of CNC can act as a nucleophile under alkaline conditions to react with epoxides, forming ether (C–O–C) linkages [89]. Thus, the etherification reaction gives the opportunity to introduce polymer-CNC covalent interaction. According to Kloser and Gray, a stable colloidal suspension of CNCs was obtained by grafting polyethylene oxide (PEO) onto the CNC surface [90]. In this case, the grafting was carried out by the reaction between the surface –OH groups of the CNC and epoxide-terminated PEO in strongly-alkaline conditions (0.37 M NaOH). The grafting reaction was successful with a yield of 85%. This grafting of a water-soluble non-ionic polymer onto the CNC surface made its suspension sterically stabilized against the coagulation (Scheme 4).

#### 3.1.3. Peptide Coupling 

**Figure 1:**
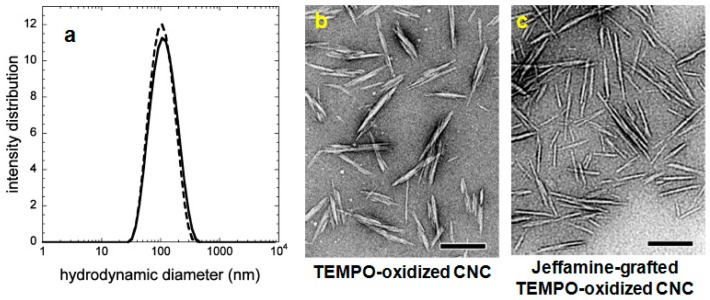
(**a**) Intensity distribution of CNC suspension (0.1 wt %) in water (solid line) and in DMF (dotted line), and TEM images of (**b**) TEMPO-oxidized CNC in water, and (**c**) sterically-stabilized Jeffamine-grafted CNC in water (reproduced with the permission from [92]).

Formation of amide bonds (–CONH) between the CNC and polymer was also found to be beneficial for the preparation of nanocomposites. In this case, it is necessary to use TEMPO-oxidized CNC containing –COOH functionalities and –NH_2_-terminated polymer [91]. Azzam and co-workers adopted this approach to graft NH_2_-terminated Jeffamine (a statistical copolymer of ethylene oxide and propylene oxide) to the TEMPO-oxidized CNC, forming a peptidic linkage between the polymer and CNC [92]. The coupling reaction was carried out using EDAC/NHS (1-ethyl-3-(3-dimethylaminopropyl)-carbodiimide)/*N*-hydroxysuccinimide) in both water and DMF suspension of TEMPO-oxidized CNC. Figure 1a shows the monomodal intensity distribution of CNC suspension in water and also in DMF, confirming good dispersion quality in both the solvents. However, the result of the peptidic coupling reaction showed a higher degree of substitution (DS) in DMF (DS = 0.1049) rather that in water (DS = 0.059), as DMF can restrict the competition between the solvent and polymer to react on the CNC surface [93]. Figure 1b,c show the TEM images of CNC and Jeffamine-grafted CNC, exhibiting no change in the morphology even after polymer grafting. This approach enabled steric stabilization of CNC in an aqueous dispersion along with the thermo-responsive property due to the presence of the grafted polymer.

Amine-end-capped poly(oligoethylene glycol) methacrylate (POEGMA) synthesized by atom transfer radical polymerization (ATRP) was grafted to the carboxylic acid-functionalized tunicate CNCs by the peptidic coupling reaction in DMF [94]. The stimuli-responsive nature of POEGMA provided the opportunity to develop the nanocomposite with tunable mechanical properties.

#### 3.1.4. Silane Coupling

Methoxy/ethoxy silanes are highly reactive to the surface –OH groups of any nanomaterial. Thus, the researchers took this opportunity to prepare polymer/CNC nanocomposites where the polymer and the CNCs are covalently attached by the silane coupling process. At first, we can consider the use of the co-monomers, like methacryloxypropyl trimethoxysilane (MPMS) and methacryloxypropyl triethoxysilane (MPES), which are able to act as coupling agents to CNC [95,96]. These co-monomers took part in the polymerization of styrene (St) and 2-ethylhexyl acrylate (EHA) in the presence of CNC, producing a P(St-*co*-EHA)/CNC nanocomposite. Generally, under aqueous conditions, ethoxysilane functionalities are readily hydrolyzed, producing silanol (Si–OH) groups that remained attached to CNC via hydrogen bonding with surface –OH groups. Upon evaporation of water, the condensation took place and the covalent linkage between the silane compound and CNC was formed. In the prepared nanocomposite, the bonding between the polymer matrix, P(St-*co*-EHA), and CNC existed through the MPMS moiety as confirmed by the solid state ^29^Si NMR analysis. 

According to Rahmat and co-workers, vinyltrimethoxysilane (VTMS) was successfully used as a silane-coupling agent to prepare a PLA/CNC composite [97]. In this case, VTMS was first grafted onto PLA by melt-mixing at 190 °C in the presence of a free radical initiator, dicumyl peroxide (DCP). The VTMS-modified PLA was then dissolved to the dispersion of CNC in DMF/chloroform (1:3 *v*/*v*) and the mixture was subjected to electrospinning, producing PLA-*g*-VTMS/CNC nanocomposite fibers. The hydrolysis and condensation reaction of silane functionalities were performed by dipping the nanocomposite fibers in hot water (70 °C). The covalent interaction between CNC and PLA through the grafted VTMS moieties was found to be reflected in the increasing crystallinity of the nanocomposite due to the good nucleation effect of CNC. The composite showed excellent biocompatibility and good mechanical properties. 

**Figure 2:**
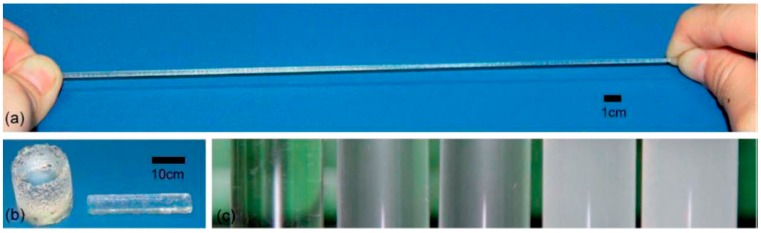
Photographs of PAA/CNC nanocomposite hydrogels, (**a**) stretched, (**b**) monolith and strip, and (**c**) transparency of gels prepared in glass tubes with different loadings of CNC (0, 0.25, 0.4, 0.65, 0.8 wt %, from left to right) (reproduced with the permission from [98]).

**Scheme 5:**
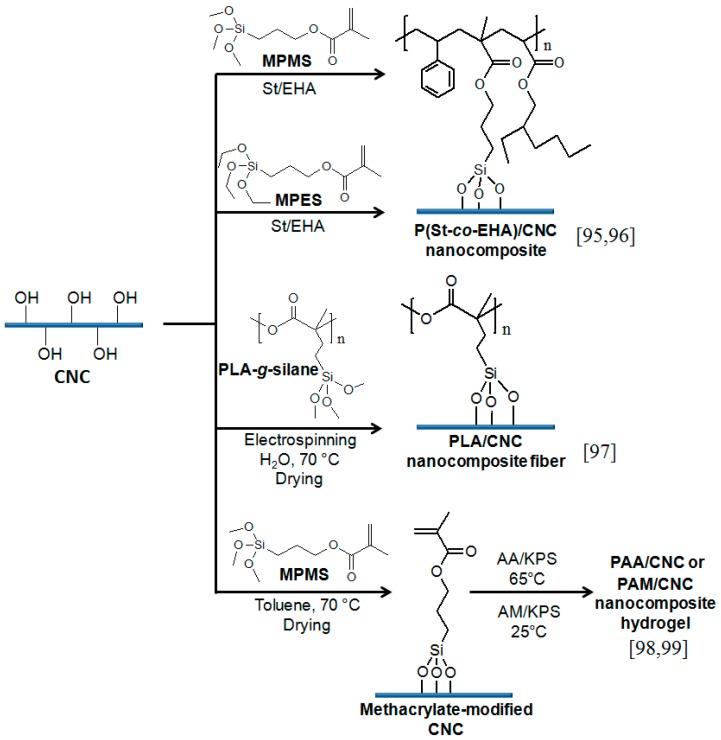
Examples of silane coupling reaction with CNC to prepare the polymer/CNC nanocomposites.

The report by Yang et al. deals with the surface modification of CNC by MPMS via the silane-coupling reaction [98]. In this case, the MPMS was added to the dispersion of CNC in toluene and the mixture was heated to 100 °C. The reaction was continued for 6 h and the MPMS-modified CNC was collected by filtration, followed by drying at 110 °C under vacuum. The successful surface modification of CNC was confirmed by FT-IR analysis, showing the absorption for Si–C (780 cm^−1^), C=C (1630 cm^−1^) and C=O (1730 cm^−1^) bonds. The modified CNC containing a polymerizable methacrylate group on the surface was employed in the polymerization of acrylic acid (AA) to produce a PAA/CNC composite hydrogel. This is how CNC was used as a cross-linker to produce the nanocomposite hydrogel having a very high elongation ratio (>1100%) (Figure 2) and high tensile strength (>350 kPa). A very similar procedure was adopted for the preparation of polyacrylamide (PAM)/CNC nanocomposite hydrogel using MPMS-modified CNC [99]. The presence of modified CNCs provided up to a two-fold increase in the elastic modulus of the nanocomposite hydrogel. Scheme 5 summarizes the examples of silane-coupling reactions utilized for the preparation of polymer/CNC nanocomposites having the nature of fiber, hydrogel, etc.

#### 3.1.5. Click Reaction

**Scheme 6:**
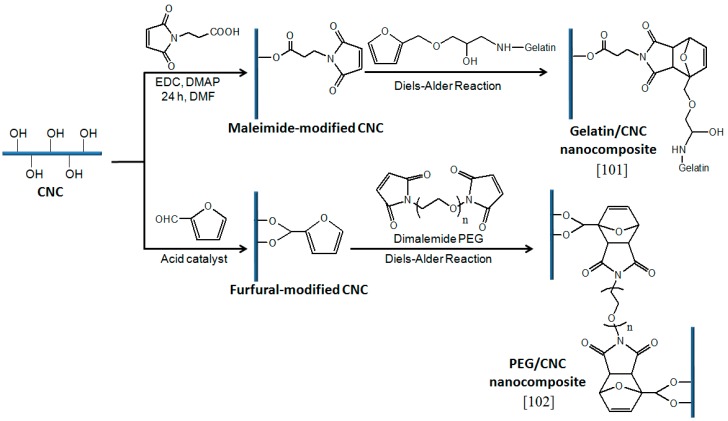
Examples of Diels-Alder click reaction on CNC to prepare self-healing polymer/CNC nanocomposites.

The group of reactions having the characteristic of high efficiency, high selectivity, and high yield without any side products, are collectively called ‘click reaction’ (Scheme 6) [100]. In this family, the Diels–Alder (DA) reaction, the cyclo-addition between a diene and a dienophile, is one of the most well-known reactions in polymer chemistry. Due to the reversible nature, this reaction is widely used for the development of self-healing polymeric materials. 

In the case of the cellulosic nanomaterials, the DA reaction was successfully used for the crosslinking in gelatine/CNC composite hydrogel [101]. In this case, the DA reaction took place between the maleimide-functionalized CNCs and furan-end-capped gelatine, producing the crosslinked nanocomposite hydrogel. However, the authors did not investigate the self-healing nature of the hydrogel. Recently, Shao et al. reported the preparation of a self-healing nanocomposite hydrogel composed of PEG and CNC [102]. In this case, furyl-modified CNCs took part in the DA reaction with maleimide-terminated PEG in the temperature range of 37–77 °C. There was a linear decrease in the gelation time with the increase in temperature. The authors demonstrated the self-healing nature of the hydrogel by visual inspection where the two fracture surfaces fused into each other when they were brought in contact and incubated at 90 °C. 

#### 3.1.6. Surface-Initiated Radical Polymerization

**Scheme 7:**
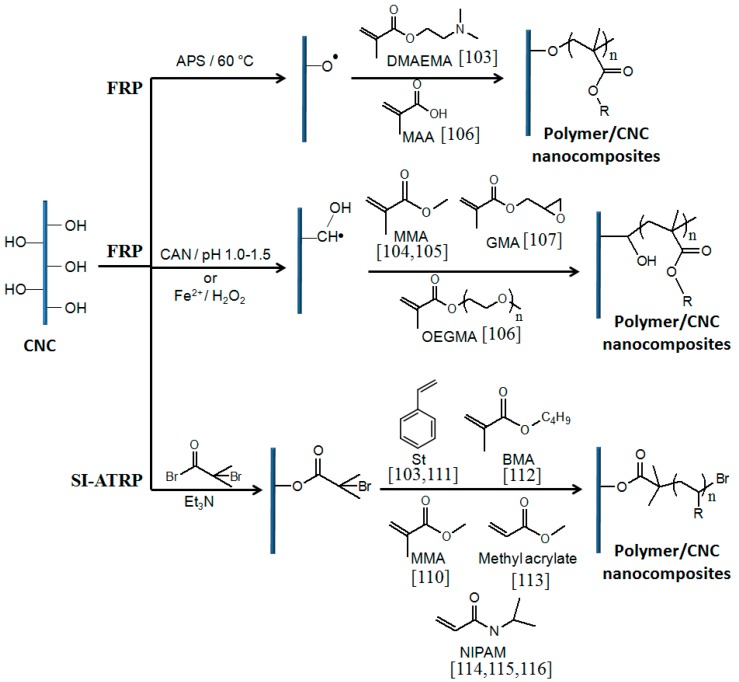
Approaches for the CNC surface-initiated FRP and ATRP to prepare polymer/CNC nanocomposites.

Free radical polymerization (FRP) is the most common and useful part of the addition polymerization process. It needs the presence of a free-radical initiator capable to produce free radicals from which the polymerization takes place. CNC was also successfully used as the initiation site by creating free radicals on its surface, thereby providing the scope for the preparation of polymer/CNC nanocomposites through the surface-initiated polymerization process. The generation of initiation sites on the CNC surface is generally done by thermal or redox initiators (Scheme 7). 

Tang et al. performed the polymerization of 2-(dimethylamino)ethyl methacrylate (DMAEMA) on CNC surface by using ammonium persulfate (APS) as the thermal initiator [103]. In this case, APS dissociates at a higher temperature (60 °C) to produce sulfate anion radicals that abstract H–atoms from the surface –OH groups of CNC. As a result, the free radicals are formed on the CNC surface and the polymerization of the DMAEMA monomer takes place from there. Thus, CNC-*g*-PDMAEMA was obtained, which was further used as a Pickering stabilizer for a heptane-in-water emulsion. In this case, the Pickering emulsion showed pH and thermo-responsive behaviour. 

Some authors demonstrated the redox-initiated polymerization of methyl methacrylate (MMA) on the CNC surface to prepare CNC-*g*-PMMA [104,105]. In this case, different redox initiators, like Fenton’s reagent (Fe^2+^/H_2_O_2_) and ceric ammonium nitrate (CAN), were successfully used to create initiation sites on the CNC surface and subsequent polymerization of MMA was carried out in water. Recently, Tang and co-workers reported the preparation of polymer/CNC nanocomposite composed of POEGMA and poly(methacrylic acid) (PMAA) grown on the CNC surface [106]. Here, they adopted a similar procedure to polymerize OEGMA and MAA monomers consecutively on the CNC surface by using CAN and APS as initiators, respectively. The composite was efficiently used as a pH-responsive Pickering stabilizer for an oil-in-water emulsion. The polymerization of glycidyl methacrylate (GMA) from the CNC surface was also performed using CAN [107]. The obtained PGMA-modified CNC was further used for the preparation of nanocomposites with PLA. 

A similar free-radical grafting process was successfully used by Zhou et al. for the preparation of PAM/CNC nanocomposite hydrogel [108]. In this case, free radicals were generated on the CNC surface by a potassium persulfate (KPS)/sodium bisulfite (SBS) redox initiator. The hydrogel was produced during the in situ FRP of acrylamide (AM) in the presence of the cross-linker *N*,*N*-methylenebisacrylamide (NMBA).

Since the inception of reversible-deactivation radical polymerization (RDRP) in the 1990s, it has been widely used to synthesize well-defined polymers because of its important features, like simplicity, livingness, robustness, etc. Apart from those unique features, this process was also found to be efficient to grow polymer chains on a particular surface with better control in molecular weight and dispersity. This method is known as surface-initiated RDRP (SI-RDRP). In this case, initiator molecules or chain transfer agents are generally attached onto the nanoparticles by utilizing their surface –OH groups. Then, the controlled polymerization of vinyl monomer is performed to produce polymer nanocomposites. This process can be successfully applied to CNCs via the surface-initiated atom transfer radical polymerization (SI-ATRP) process to prepare polymer/CNC nanocomposites having chemical bonding between the polymer and CNCs [109]. 

In this context, Yu et al. performed the functionalization of CNC by 2-bromoisobutyryl bromide (BiB) and subsequently copolymerized MMA and BA to produce CNC-*g*-P(MMA-*co*-BA) [110]. The modified CNC was used for the reinforcement of P(MMA-*co*-BA). A similar functionalization of CNC was carried out to perform the SI-ATRP of St, producing CNC-*g*-PSt [111]. The modified CNC was compounded with PMMA by solution casting to prepare PMMA/CNC nanocomposite. The SI-ATRP of butyl methacrylate (BMA) from the CNC surface was performed by Boujemaoui et al. [112]. The CNC-*g*-PBMA was used for the reinforcement of PCL matrix. Hatton and co-workers reported the photo-induced SI-ATRP of methyl acrylate on CNC functionalized with BiB [113]. The grafted polymers had a controlled molecular weight and narrow dispersity (≥1.1). 

Single-electron transfer living radical polymerization (SET-LRP) was carried out by Zoppe and co-workers to graft poly(*N*-isopropylacrylamide) (PNIPAM) from the CNC surface [114,115]. It gives the opportunity to improve the stability of CNC dispersion via steric stabilization provided by the grafted polymer chains. It also provides a platform to fabricate a thermo-responsive material based on nanocellulose. CNC-*g*-PNIPAM was also successfully used as a Pickering stabilizer for an oil-in-water emulsion [116]. In this case, the Pickering emulsion was very much stable at room temperature for four months. However, the emulsion became unstable when it was heated at temperatures higher than the LCST of PNIPAM. Thus, the Pickering emulsion showed thermo-responsive behaviour due to the presence of PNIPAM. 

#### 3.1.7. Ring-Opening Polymerization 

Ring-opening polymerization (ROP) is a very useful polymerization technique. It is widely used for the production of many industrially-important polymers, like nylon 6, polynorbornene, polyphosphazene, polyethylene oxide, etc [117]. Researchers tried to use CNC as a reinforcing filler for the polymers prepared via ROP. It was found that the –OH groups of CNC can be used as the initiating species for the ROP [39]. In this regard, we found reports on the preparation of CNC-*g*-poly(L-lactide) (PLLA) by the ROP of *L*-lactide initiated from the CNC surface [118,119,120]. A similar approach was adopted by Wu et al. to prepare CNC-*g*-poly(*D*-lactide) (PDLA) [121]. The polymerizations were carried out in the presence of tin(II)ethyl hexanoate [Sn(oct)_2_] or magnesium hydride as a catalyst. The prepared CNC-*g*-PLLA was further used to prepare a composite with PLA or PLLA, whereas CNC-*g*-PDLA was compounded with PLLA. All the nanocomposites exhibited improved mechanical, thermal, and barrier properties. In addition, Chen et al. used CNC-*g*-PLLA as an antinuleation agent to retard the crystallization of poly(3-hydroxybutyrate) (PHB), a biodegradable aliphatic polyester [122]. Since unmodified CNC does not have such a retardant effect, but rather promotes the crystallization of PHB, they argue that these nanomaterials allow the control of the crystallization behaviour.

**Scheme 8:**
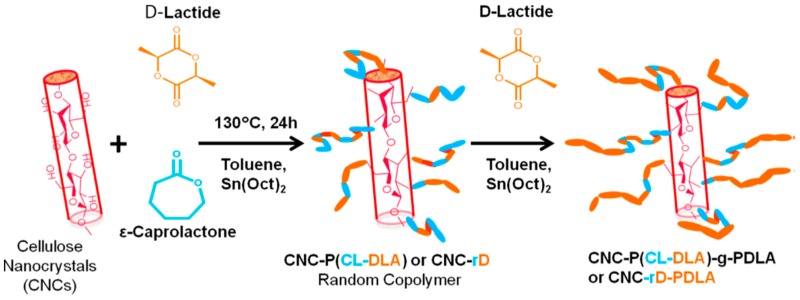
Functionalization of CNC by ROP of *D*-lactide and ɛ-caprolactone (reproduced with the permission from [123]).

Muiruri et al. performed the ring-opening copolymeriation of *D*-lactide and ɛ-caprolactone to produce CNC-*g*-P(CL-DLA), followed by the further polymerization of *D*-lactide to finally obtain CNC-*g*-P(CL-DLA)-PDLA (Scheme 8) [123]. The nanocomposite of commercial PLLA and the modified CNC was prepared by the solution mixing in chloroform. The reinforcement by modified CNC accounted for the 20-fold increase in the strain at break.

#### 3.1.8. Esterification

CNC can take part in the polymerization not only as the initiator, but also as monomer, by carrying polymerizable units. CNCs can be converted to monomers by incorporating some polymerizable groups on their surface, as already shown in silane-coupling reaction. The esterification process was also found to be an easy and widely-used technique for the surface modification of CNCs [124]. Mannan and co-workers employed the reaction between acid chloride and surface –OH groups to modify the CNC surface [125]. In this case, 10-undecenoyl chloride was used to create polymerizable alkyl vinyl moieties on the CNC surface. Finally, the polymerization of ethylene was carried out in presence of the modified CNCs. This investigation provides a route to prepare polyethylene (PE)/CNC composites with better compatibilization between non-polar PE and polar CNC. However, the dispersion of CNCs in the PE matrix was not well explored in that report.

Apart from the only surface modification of CNCs, the esterification between CNC and the polymer matrix was also successfully carried out. Goetz et al. reported the esterification reaction between the carboxylic acid groups of poly(methyl vinyl ether-*co*-maleic acid) (PMVEMA) and the surface hydroxyl groups of CNC to produce a PMVEMA/CNC nanocomposite having a very high (up to 900%) water absorption capacity [126]. The esterification reaction between –OH groups of CNC and maleic anhydride (MA) units of MA-grafted poly(butylene adipate-*co*-terephthalate) (PBAT) was expected to happen during the preparation of the PBAT/CNC nanocomposite by the reactive extrusion process [127]. The nanocomposite exhibited more than a 100% increase in the Young’s modulus compared to the pristine PBAT. We can also find a similar approach to use MA-grafted polypropylene (PP) (MAPP) as the coupling agent to CNC for the preparation of PP/CNC nanocomposite [128].

### 3.2. Polymer/CNC Nanocomposites with Non-Covalent Interactions

#### 3.2.1. Hydrogen Bonding

Due to the presence of abundant –OH groups on the CNC surface, many researchers utilized the H-bonding interaction between polymer and CNC to prepare CNC-based nanocomposites. It has been found to be successful approach to produce nanocomposites with many important properties. 

Zhu and co-workers utilized the H-bonding interaction between 2-ureido-4-pyrimidinone (UPy) moieties and CNC to develop a nanocomposite based on the copolymer of OEGMA and methacrylate derivative of UPy (UPy-MA) [129]. UPy is considered as a good H-bonding donor and acceptor. In this case, the nanocomposite exhibited a highly-ordered orientation of CNCs due to the effective polymer-CNC interaction. The polymer–filler H-bonding interaction was observed in PMMA/CNC nanocomposite prepared by solution casting in acetone [130]. In the nanocomposite, there was 260% increase in storage modulus at the glass transition temperature only at 0.5 wt % CNC content. In this case, the H-bonding interaction between the –OH groups of CNCs and >C=O groups of PMMA was reported to be responsible for the improvement of thermal and mechanical properties in the nanocomposite.

Dhar et al. identified the H-bonding interaction between PLA and CNC using FT-IR spectra [131]. The shift in carbonyl peak from 1746 cm^−1^ to 1742 cm^−1^, C–O–C stretching from 1180 cm^−1^ to 1176 cm^−1^ and free hydroxyl (C–OH side groups) from 1128 cm^−1^ to 1126 cm^−1^ of PLA suggested the possible H-bonding interactions. The nanocomposite exhibited up to 1.75-fold increase in the storage modulus with respect to the pristine PLA as measured over a temperature range of less than 60 °C, which is the *T*_g_ of PLA. 

We can mention poly(vinyl alcohol) (PVA) as one of the important polymers providing H-bonding interaction with the CNCs. An aqueous mixing process was reported for the preparation of PVA/CNC nanocomposite even at high CNC loading, up to 67 wt %. [132]. According to Nassima El Miri and co-workers, PVA–chitosan blend was also found to get reinforced by the H-bonding interaction with CNC [133]. The obtained bio-nanocomposite film had a 77% increase in tensile strength in the presence of 5 wt % CNC. The reinforcement of PVA by the mixture of CNC and graphene oxide was reported by the same author [134]. The nanocomposite exhibited 124% increase in tensile strength due to the extensive H-bonding interaction PVA and the hybrid nanofiller. Orr and Shofner reported the polymer–filler H-bonding interaction in polyethyle-*co*-vinyl alcohol/CNC nanocomposite [135]. The interaction was also found to be responsible for the reinforcement of the PVA/carboxymethyl cellulose (CMC) blend matrix by CNC [136]. At 5 wt % CNC content, the PVA/CMC/CNC nanocomposite showed an 83% increment in tensile strength along with an 87% decrease in water vapour permeability.

H-bonding interaction was successfully utilized to prepare polyethylene glycol (PEG)/CNC nanocomposites [137,138] and hydrogel [139,140]. The nanocomposites were prepared by casting the aqueous suspensions, whereas the hydrogels were obtained by the polymerization of PEG-diacrylate (PEGDA) in the presence of CNC. In this case, the >C=O and other O atoms of PEGDA strongly interacted to the surface –OH groups of CNC through H-bonding. The same interaction among poly(propylene carbonate) (PPC), PEG, and CNC in a PPC/PEG/CNC nanocomposite created a three-dimensional network [141]. As a result, the oxygen permeability of the nanocomposite was reduced by 76.1% in the presence of only 0.3 wt % CNC. The H-bonding interaction between ethylene glycol moiety and CNC was also utilized for the fabrication of poly(oligoethylene glycol methacrylate) (POEGMA)/CNC nanocomposite hydrogel [142]. In the presence of 5 wt % CNC, there was an increase in storage moduli of up to 35-fold as observed from the rheological measurements of the hydrogels at 22 °C, along with a faster gelation rate in the nanocomposite. 

Kelly and co-workers reported the preparation of PAM/CNC photonic hydrogel which showed a colour change in the presence of external stimuli, like solvent, pH, and temperature [143]. In the hydrogel, due to the evaporation-induced self-assembly (EISA) the CNCs formed a chiral nematic phase with a helical pitch at visible wavelengths. As a result, the hydrogels exhibited iridescence, which was fully reversible between dry and swollen states, corroborating the influence of strong H-bonding interaction between CNC and PAM. It is also possible to observe the structural colour in PEG/CNC nanocomposites upon slow drying, which facilitates the chiral nematic orientation of CNC [144]. According to Gu et al., a similar iridescence was also observed for the PEG/CNC nanocomposite upon EISA [145]. There was H-bonding interaction between PEG and CNC as evidenced from the shifting of the FT-IR absorption band at 3300 cm^−1^ for the CNC hydroxyl groups to a higher frequency in the nanocomposite. 

**Scheme 9:**
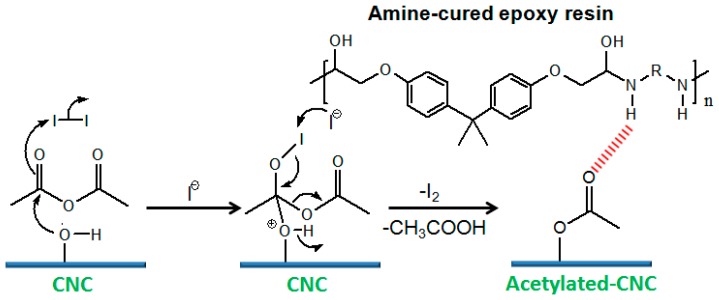
*I*_2_-catalyzed solvent-free acetylation of CNC and reinforcement of amine-cured epoxy resin through hydrogen-bonding interaction (adopted from [146]).

H-bonding interaction was also reported in acetylated CNC/epoxy nanocomposite [146]. In this case, the acetylated CNC with a DS of 2.18 was prepared by a one-step, iodine-catalyzed, and solvent-free acetylation reaction (Scheme 9). In the nanocomposite, there exists an H-bonding interaction between the >C=O of acetylated-CNC and the –N–H/–O–H of the amine-cured epoxy resin, as evidenced from the considerable shift in stretching band of hydrogen bonded amino groups from 3400 cm^−1^ to 3280 cm^−1^. However, this acetyl DS of 2.18 seems to be too high to retain cellulose I crystals detected by the authors; further detailed characterization is expected. According to Xu et al., acetylated CNC (DS up to 2.71) reinforced PCL via H-bonding interaction [147]. As they have found, the acetylated CNCs with a DS of 2.36 and 2.71 could not maintain the form of nanocrystals and the acetylated products were amorphous. The researchers interpreted that the continuous decrease in crystallization temperature (*T*_c_) of the nanocomposite with the increase in the DS of acetylated-CNC was indicative of the H-bonding interaction between PCL and surface amorphous molecules of acetylated CNCs. 

It is reasonable to use aqueous latex from the viewpoint that the solvent exchange is unnecessary in principle for the preparation of nanocomposites and, consequently, the process is easy. Mariano et al. prepared an oxidized natural rubber (ONR)/CNC nanocomposite by casting and evaporation of the aqueous suspension. The H-bonding interaction between the –OH groups of both ONR and CNC gave rise to the improved mechanical and thermal properties in the nanocomposite [148]. The epoxidized natural rubber (ENR)/CNC nanocomposite exhibited reversible water-responsive behaviour due to the H-bonding interaction between the –OH groups of the CNC and the epoxy units of ENR [149]. With the increase in the CNC content, there was a drastic increase in the storage modulus of ENR/CNC nanocomposite at 37 °C compared to the NR/CNC nanocomposite. H-bonding interaction was found to be operating for the reinforcement of water-borne polyurethane (WPU)/CNC nanocomposites prepared by solution casting [150]. From the FT-IR analysis, the polymer–filler interaction was identified by the appearance of shoulder peak at 1690 cm^−1^ for the hydrogen-bonded >C=O groups of WPU along with the peak at 1722 cm^−1^ due to the free >C=O groups. The nanocomposite films exhibited improved thermo-mechanical properties, as well as a higher water absorption capacity.

Song et al. demonstrated the fabrication of a supramolecular polymer/CNC nanocomposite based on hydroxyl- and carboxyl-functionalized soybean oil-derived polymers [151]. The nanocomposite was prepared by solution casting from DMF. A supramolecular network was formed by the extensive H-bonding between the –OH groups of CNC and –OH/–COOH functionalities of the soy polymer. The nanocomposite not only exhibited improved mechanical properties, but also had a fast and reversible mechanical response to water.

**Scheme 10:**
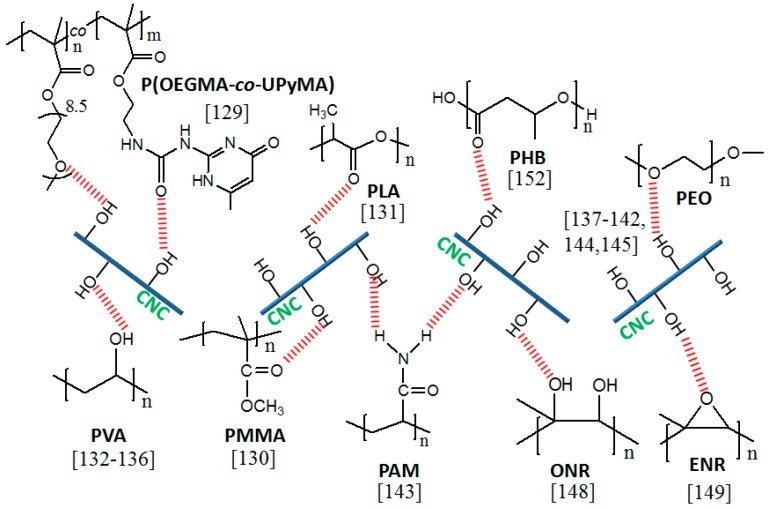
Examples of polymers having hydrogen bonding interaction with CNC in their corresponding nanocomposites.

Seoane and co-workers reported the preparation of a PHB/CNC bio-nanocomposite for packaging applications [152]. In this case, CNC was responsible to reinforce PHB by the H-bonding interaction between >C=O of PHB and –OH of CNC as evidenced from the FT-IR analysis. The nanocomposite attained improved mechanical and barrier properties at optimized concentrations of CNC (6 wt %), making it suitable to replace polypropylene for packaging applications. When the nanohybrid of CNC and ZnO was used for the reinforcement of poly(3-hydroxybutyrate-*co*-3-hydroxyvalerate) (PHBV) there was an H-bonding interaction between the –OH groups of CNC and ester carbonyl groups of PHBV [153]. Such an interaction affected the crystallinity in the nanocomposite, and significantly improved the thermal and mechanical properties. Scheme 10 shows the chemical structures of the polymers reinforced by CNC through H-bonding interaction.

#### 3.2.2. Electrostatic Interaction

CNC obtained from the sulphuric acid hydrolysis of native cellulose contains sulfate ester groups on its surface. As a result, it attains a negative charge on its surface. Thus, the fabrication of nanocomposites using cationic polymers can be considered as a fertile approach. Mabrouk et al. prepared the PBMA/CNC nanocomposite using the electrostatic interaction between PBMA and CNC [154]. In this case, cationic-PBMA was obtained by the miniemulsion polymerization of butyl methacrylate in the presence of a cationic surfactant named dodecylpyridinium chloride. Thus, the presence of negatively-charged CNC during the miniemulsion polymerization initiated the electrostatic interaction to attach to the PBMA particles, producing the nanocomposite which showed a good dispersion of CNC in the PBMA matrix. In addition to the native electrostatic interaction between the polymer and CNC, it is also possible to control the orientation and dispersibility of CNCs in the polymer matrix by applying an external electric field [155]. Ten and co-workers adopted such an approach in preparing PHBV/CNC nanocomposites by the solution mixing process [156]. In this case, the nanocomposite was prepared by the evaporation of solvent DMF under an external DC electric field which regulated the orientation of the CNCs in the PHBV matrix. The presence of field-induced dipolar interaction between the polymer and the CNC contributed to the improved mechanical properties in the prepared nanocomposite. According to Vollick et al., a cationic copolymer latex prepared by the emulsion copolymerization of butyl acrylate (BA), 9-vinylanthracene, and 2-(methacryloyloxy)ethyl acetoacetate, had strong electrostatic interactions with the negatively-charged CNCs, producing a stable colloidal gel [157].

#### 3.2.3. Physisorption

Cellulose derivatives can be physically adsorbed onto nanocellulose [158]. Many researchers took this opportunity to produce nanocomposites based on cellulose derivatives. McKee and co-workers prepared methyl cellulose (MC)/CNC nanocomposite hydrogel which exhibited thermo-responsive behaviour [159]. CNCs were found to be nicely dispersed in MC-matrix as observed from the cryo-TEM analysis. Moreover, at 20 °C the storage modulus of the nanocomposite hydrogel can be tuned from 1.0 to 75.0 Pa upon increasing the CNC content from 0 to 3.5 wt %. 

**Scheme 11:**
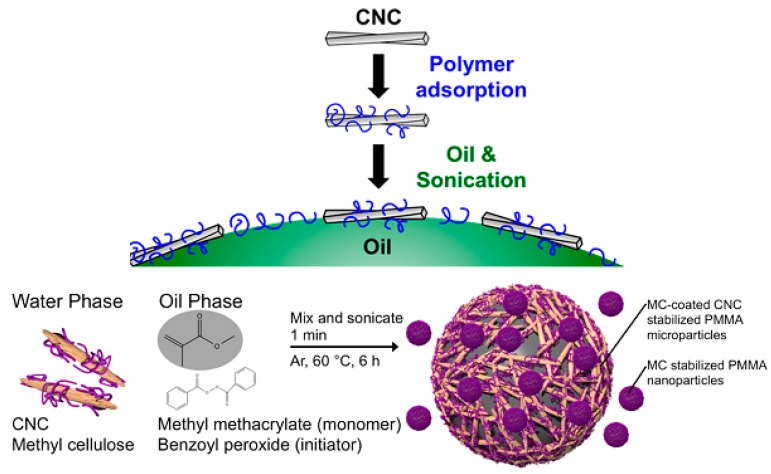
Pickering stabilization of O/W emulsion by MC or HEC-adsorbed CNC and the subsequent polymerization of MMA as the oil phase to obtain CNC-coated, along with the MC-stabilized, PMMA particles (reproduced with the permission from [160,162]).

The nanocomposites based on MC and hydroxyethyl cellulose (HEC) were successfully used as Pickering stabilizers for oil-in-water emulsions (Scheme 11) [160,161,162]. Long-term emulsion stability was achieved by the synergistic stabilization effect of CNC and the cellulose derivatives, which cannot be obtained using CNC alone. Moreover, with the addition of tannic acid (TA), this type of emulsion stabilization system provides the opportunity to re-disperse the dried emulsion due to the complexation between TA and the cellulose derivative. Using MMA as the oil phase in the Pickering emulsion, the CNC-coated PMMA microspheres can be obtained after free radical polymerization (Scheme 11). 

Apart from the emulsion stabilization, the MC/CNC nanocomposite was also found to produce very stable aqueous foams [163]. The lowest density foam was obtained using the mixture of 2 wt % CNC and 0.5 wt % MC. The foams are capable of withstanding temperatures up to 70 °C. Oun and Rhim reported a CMC/CNC nanocomposite with the desired improvement in mechanical and barrier properties [164]. Up to a 45.7% increase in tensile strength and up to a 26.3% decrease in water vapour permeability in the nanocomposite at 5 wt % of CNC was observed. The blend of CMC and starch was also found to be reinforced by CNC, producing an eco-friendly bio-nanocomposite film for packaging applications [165]. The presence of CNC increased the tensile strength up to 65% along with the reduction in water vapour permeability. 

Cellulose acetate (CA)/CNC nanocomposite prepared by melt extrusion with a plasticizer (triethyl citrate) also had improved mechanical properties (a 14% increase in the Young’s modulus) when the predispersion of CNC in the CA solution was performed prior to the melt extrusion [166]. The predispersion method was found to also be better in terms of the distribution of CNC in the CA matrix compared to the direct mixing method. 

Boujemaoui et al. prepared amphiphilic block copolymers, such as PDMAEMA-*b*-PBMA and P(DMAEMA-*co*-MAA)-*b*-PBMA, via ATRP and reversible addition-fragmentation chain transfer (RAFT) polymerization, respectively, and performed the surface modification of CNC by the physical adsorption of those block copolymers onto the CNC surface [112]. The modified CNCs were used for the reinforcement of the PCL matrix.

#### 3.2.4. Compatibilization through Surface Modification

Simple surface modification of CNCs was found to be beneficial for the preparation of polymer nanocomposites. In most cases, the surface modifier significantly contributed to improving the interfacial adhesion between the polymer and CNC. Table 1 summarizes the type of surface modifier and the result of reinforcement in the respective polymer matrix. 

It is possible to perform the surface modification of CNC by the ion-exchange process in order to improve its dispersibility in the polymer matrix. The sodium ions on sulfated CNCs can undergo this ion exchange with alkylammonium ions [167]. Fox et al. modified CNCs by using ionic liquid cations, like methyl(triphenyl)phosphonium (MePh_3_P^+^), 1,2,3-trimethylimidazolium (Me_3_Im^+^), 1-hexyl-2,3-dimethylimidazolium (HxMe_2_Im^+^), and 1-hexadecyl-2,3-dimethylimidazolium (HdMe_2_Im^+^) [168]. The modified CNCs attained good dispersion in both epoxy and PS matrix. However, there was no significant improvement in mechanical properties in addition to the 10% increase in water absorption. Mariano and co-workers reported the surface modification of CNC by imidazolium-end-capped PLLA for the reinforcement of PLA [169]. However, there was not so much improvement in the tensile properties. 

The modification of CNC by quaternary ammonium cations bearing long alkyl chains was also found to be useful to reinforce polypropylene (PP) [170]. In this case, the water-based modification of CNC was carried out by hexadecyltrimethylammonium bromide (QS). The PP/CNC nanocomposite was obtained through the melt extrusion process. The nanocomposite exhibited improved mechanical properties, as observed in the eight-fold increase in elongation at break compared to the pristine PP. Kaboorani et al. used the same CNC–modification process for the reinforcement of UV-curable acrylic coating for wood [171]. The nanocomposite coating had a significant improvement in barrier (lower water vapour transmission) and optical properties (higher optical clarity) in the presence of up to 3 wt % modified CNC. 

PEG was found to act as a good compatibilizer in HDPE/CNC nanocomposite [172]. The PEG-adsorbed CNCs were prepared by the aqueous mixing of PEG and CNC followed by freeze drying. The nanocomposite, obtained by melt compounding at 160 °C not only exhibited higher crystallinity, but also had improved mechanical properties at an optimum CNC content of 1.5 wt %. Azouz and co-workers developed PEG/CNC nanocomposite by utilizing the affinity of PEG chains to be adsorbed on the CNC surface [173]. The modified CNC was successfully used for the reinforcement of hydrophobic polymer matrices, like low-density polyethylene (LDPE). A similar phenomenon was also observed in the surface modification of CNC by PEG-*b*-PLLA [169]. The modified CNC was used for the reinforcement of the PLA matrix. The surface modification of CNC by Beycostat, a PEG-based surfactant, was also found to be beneficial for the reinforcement of PLA [174], as well as poly(butylene/triethyleneglycol succinate) random copolymer [175]. Meesorn et al. used PVA/CNC nanocomposite for the reinforcement of a poly(ethyleneoxide-*co*-epichlorohydrin) (EO-EPI) matrix [176]. The EO-EPI/PVA/CNC nanocomposite was prepared by solution casting from DMSO. In this case, PVA, having H-bonding interaction with CNC, acted as compatibilizer between EO-EPI and CNC. The nanocomposite had an increase in tensile strength of about four-fold in the presence of PVA. A similar approach was adopted by Ludueña et al. to reinforce polybutylene-succinate (PBS) using PEG-modified CNC [177]. However, in this case, the nanocomposite did not exhibit a significant improvement in mechanical properties. There was only a slight increase in the elongation at break, although the dispersion of CNC was claimed as homogeneous. 

The urethanization reaction, as discussed earlier, was not only used for the covalent coupling between polymers and CNC, but also utilized for the surface modification of CNC to improve its compatibility to the polymer matrix. The surface modification of CNC by *n*-octadecyl isocyanate was performed to improve the dispersion of CNC in a PLA/NR blend matrix [178,179]. The appearance of a new absorption at 1704 cm^−1^ in FT-IR confirmed the formation of carbamate (–NHCOO–) bonds between CNC and the long alkyl chains (C18). However, in the presence of C18-modified CNC the mechanical properties of the PLA/NR blend deteriorated significantly. Morelli et al. performed the CNC surface modification by phenylbutyl isocyanate in the presence of dibutyltin dilaurate (DBTDL) in toluene at 110 °C, forming urethane linkages [180]. The modified CNC was used for the preparation of nanocomposites with PBAT by melt extrusion. The bio-degradable PBAT/CNC nanocomposite not only exhibited improved mechanical and thermal properties, but also had much lower water vapour permeability. 

The surface modification of CNC by coumarin-NCO was found to be an interesting approach due to the dimerization reaction of coumarin under UV radiation [181]. In this case, the CNC modification reaction was carried out using DBTDL in DMF at 100 °C. The nanocomposite of EO-EPI was fabricated using the modified CNC via solution casting in DMF. The nanocomposite can undergo crosslinking by UV radiation due to the dimerization of coumarin fragments on CNCs. The same research group developed a UV light-healable nanocomposite material based on the UPy-modified CNCs [182]. In this case, the surface modification of CNC was carried out using NCO-terminated ureidopyrimidone (UPy), which was attached to CNC’s surface via the reaction between –NCO and surface –OH groups of CNC catalyzed by DBTDL in DMF at 100 °C. The nanocomposite of UPy-functionalized poly(ethylene-*co*-butylene) and UPy-modified CNC was obtained by solution casting in THF. In the nanocomposite, there was an H-bonding interaction between the UPy-moieties attached to the polymer and the UPy-moieties on CNC leading to the 12-fold increase in the Young’s modulus at 20 wt % modified-CNC content. The polymer-CNC interaction was found to be sensitive toward ultraviolet radiation, making the nanocomposite optically healable. A similar surface modification of CNC by UPy-NCO was performed for the reinforcement of LDPE, LLDPE, polystyrene-*b*-polybutadiene-*b*-polystyrene (SBS), and EO-EPI [183]. All the nanocomposites exhibited improved stiffness and strength.

Imato et al. performed the surface modification of CNC by urethanization using NCO–terminated diarylbibenzofuranone (DABBF) [184]. The urethanization reaction was catalyzed by DBTDL and was performed at 40 °C for 72 h. A DABBF-based crosslinked polymer was used as the matrix for the preparation of nanocomposite using the modified CNC via solution casting from DMF. In this case, DABBF moieties acted as mechanophores due to their ability to change the colour under mechanical stress. They also offered reversible covalent bonding between the polymer and CNC, providing the self-healing property to the nanocomposite. 

There are examples of surface modification of CNC by the silane-coupling reaction using *N*-(β-aminoethyl)-ɣ-aminopropyltrimethoxysilane [185] and (3-aminopropyl) triethoxysilane [186] and (3-aminopropyl) trimethoxysilane [187]. The modified-CNCs were used for the reinforcement of unsaturated polyester resin (UPR), polyimide (PI), and epoxy resin, respectively. The UPR/CNC nanocomposite had only 20% increase in tensile strength at 2 wt % filler loading, while about 40% improvement in tensile modulus was observed for PI/CNC nanocomposite at 3 wt % CNC content.

Acetylated-CNC was successfully used for the reinforcement of PLA matrix [188]. The acetylation reaction was carried out by acetic anhydride in the presence of pyridine at 105 °C for 15 h. The acetylation of CNC by acetic anhydride was also performed by Zhang et al. for the reinforcement of PBAT [189]. The surface modification of CNC by MA was performed to prepare the nanocomposite of PLA [190]. The nanocomposite had significant improvement in water vapour adsorption. The thermodynamic miscibility of PCL with normal alkanoic acid esters of cellulose molecules (not nanocellulose) has been systematically investigated [8,191]. In the series of studies, it was proposed that the driving forces to achieve the miscibility in the thermodynamic sense was the dipole interaction in association with the similarity of the partial structure of the component polymers; the high DS and long ester side chains are necessary for achieving the miscibility, and cellulose acetate with any DS is not compatible with PCL. Even for the discussion of the compatibility of nanocomposite systems, the authors feel that it is necessary to refer to examples of blend compatibilization at the molecular level.

Fox and co-workers performed the surface modification of TEMPO-oxidised CNC by allyl amine [192]. The modification reaction involving –COOH of CNC and –NH_2_ of allyl amine was carried out using peptide coupling chemistry by EDC/NHS to obtain the –CONH linkages. The allyl-modified CNC was used as reinforcing nanofiller for polyvinyl acetate (PVAc) matrix. Zhang and co-workers also adopted a similar approach to attach alkyl amines to the TEMPO-oxidised CNC [193]. The alkyl-modified CNCs were successfully used as a Pickering stabilizer for a styrene-in-water emulsion and the subsequent polymerization finally produced PS/CNC nanocomposite latex. Alkyl-modified CNCs were also used as a reinforcing nanofiller for an ethylene-vinyl acetate copolymer (EVA) matrix [194].

## 4. Polymer Nanocomposites Reinforced by CNF

In recent years, the development of polymer nanocomposites using CNF as a nanofiller has attained a tremendous research interest due to the exceptional properties of CNF [195]. However, similar to CNCs, there is a concern with the hydrophilicity of CNF making it incompatible with the hydrophobic polymer matrix. Thus, it is important to incorporate any sort of polymer–CNF interaction to obtain the desired nanocomposite. In this section, the authors shall discuss the different approaches to achieve the interaction between the polymer and CNF in their nanocomposites. In this case, also, the authors explain the approaches under two modes of interaction as covalent and non-covalent. 

In preparing composites with polymers, the CNF dispersion may be treated with a homogenizer. However, unlike the rod-like CNC systems, in the case of the homogenization by blades rotating at high speed, intertwining among nanofibers may be promoted. Attention is necessary for this point.

### 4.1. Polymer/CNF Nanocomposite with Covalent Interactions

#### 4.1.1. Urethanization

Numerous surface –OH groups of CNF are prone to react with –NCO functionalities present in the polymer, producing the polymer/CNF nanocomposite having polymer–filler urethane (–COONH) linkages. In one such example by Wu et al., the urethane linkages were formed between CNF and WPU during the in situ preparation of WPU in the presence of hexamethylene diisocyanate (HDI), polyol, and CNF [196]. In this case, CNF served as the crosslinker in the WPU/CNF nanocomposite showing about 100% and 86% improvement in the Young’s modulus and tensile strength, respectively.

#### 4.1.2. Silane Coupling

It is well known that methoxy/ethoxy silanes are very much prone to hydrolysis, producing silanol (Si-OH) groups. Subsequent condensation reactions with the –OH groups of any nanomaterial resulted in surface modification. Under this topic of discussion, it is worth mentioning that the recent report by Kobe et al. performed the surface modification of CNF by 3-(trimethoxysilyl)propyl methacrylate (MPS) in DMAc at 100 °C under stirring [197]. In the next step, this modified CNF containing several monomer units was used as a multifunctional crosslinker in the polymerization of NIPAM to finally produce PNIPAM hydrogel. The obtained nanocomposite hydrogel contained approx. 90% water and exhibited very good mechanical properties as it could be stretched about 700-fold without fracturing.

The silane coupling reaction was also utilized for the modification of TOCN by PE and the subsequent preparation of LLDPE/TOCN nanocomposite [198]. In this case, the PE was end-functionalized with alkoxysilane that reacted with TOCN in toluene at 110 °C. The obtained TOCN-g-PE was further blended with LLDPE in xylene at 140 °C. There was 106% increase in the Young’s modulus in the presence of 5 wt % TOCN-*g*-PE.

#### 4.1.3. Etherification

Similar to CNCs, the –OH groups of CNF was also successfully used for coupling with epoxide moieties in the polymer. Ansari and co-workers found CNF to significantly contribute to the curing of epoxy (EP)/CNF nanocomposite [199]. During the curing process, the –OH groups of the CNF reacted with the epoxy units resulting in a crosslinked nanocomposite with three-fold increase in stiffness and strength compared to the pristine nanocomposite. Moreover, there was a gradual increase in *T*_g_ with the increase in CNF content, suggesting the covalent bonding between EP and CNF. In addition, the nanocomposite exhibited considerably lower moisture adsorption. Surface-modified CNF can also be used for such similar crosslinking reactions to produce EP/CNF nanocomposite [200]. The surface modification of CNF by polyethylenimine (PEI), a branched polymer with numerous amine groups, offered the scope for the crosslinking reaction between amine and epoxy units resulting in a 237.6% increase in the Young’s modulus. Recently, Chitpong and Husson developed polyacrylic acid (PAA)/CNF nanocomposite membrane for the removal of trace amounts of Cd ions from water [201]. To prepare the nanocomposite, surface modification of CNF was carried out using polyglycidyl methacrylate (PGMA) where some epoxy units of PGMA reacted with the surface –OH groups of the CNF. The remaining unreacted epoxy units were later utilized to graft PAA. The produced cation-exchange membrane had very high Cd binding capacity (>160 mg/g) in combination with higher permeability. 

#### 4.1.4. Peptidic Coupling

**Scheme 12:**
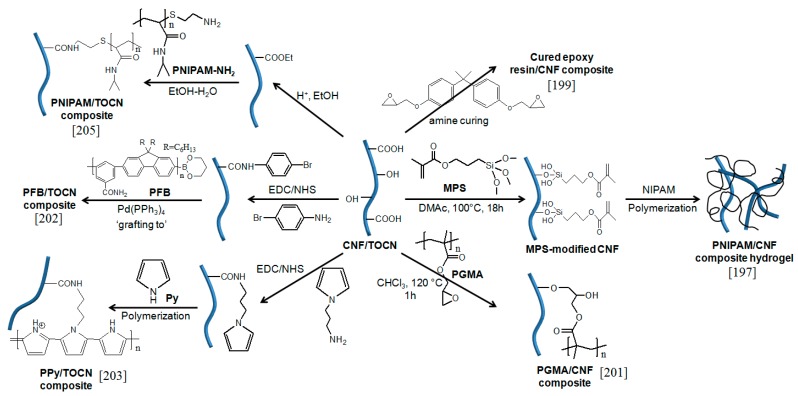
Etherification, silane coupling and peptidic coupling approaches to attach polymers onto CNF producing the polymer/CNF nanocomposites.

Due to the presence of the –COOH group at C6, TEMPO-oxidized CNF (TOCN) gives the opportunity to graft polymers onto the nanofibers via amide (–CONH) bond formation. Scheme 12 shows the peptidic coupling approach along with the other approaches to incorporate covalent interaction between the polymer and CNF. 

Niu and co-workers reported the synthetic process to graft poly(fluorene-*co*-benzamide) (PFB) onto TOCN through amide linkages [202]. In this case, the TOCN was first reacted with 4-bromoaniline, and then the Suzuki-coupling reaction was carried out to graft PFB. The reaction between the –COOH groups of TOCN and –NH_2_ units of 4-bromoaniline was carried out using EDC/NHS. The prepared PFB/TOCN nanocomposite can selectively recognize and rapidly respond towards the explosive vapor. Bideau et al. performed the attachment of pyrrole units onto TOCN by the reaction between the –COOH group of TOCN and the –NH_2_ groups from *N*-(3-aminopropyl)pyrrole [203]. Subsequent polymerization of pyrrole in the presence of those modified CNFs gave rise to a CNF-reinforced conducting polypyrrole (PPy) composite. The obtained PPy/CNF nanocomposite not only exhibited improved thermal and mechanical properties, but also had high conductivity of 20 S/cm, making it suitable as the electrode for supercapacitors, batteries, and sensor applications. It was found that amide coupling can be accomplished more easily using ester groups rather that the carboxylates [204]. Thus, Hakalahti et al. took this opportunity to graft –NH_2_-terminated PNIPAM onto the CNF containing ester functionalities [205]. The prepared PNIPAM/CNF composite was used in the fabrication of a thermo-responsive membrane for nanofiltration. 

#### 4.1.5. Esterification

The presence of –OH groups also provide the opportunity to graft polymer chains through the formation ester linkages. In fact, –OH groups are very much prone to react with anhydrides at elevated temperature. As a result, this process has been widely used for the modification of several cellulose derivatives [206]. 

The CNF can be turned into a multifunctional crosslinker through surface modification by MA [207]. In this case, the reaction between MA and CNF was carried out in DMAc at 120 °C for 3 h. The obtained MA-modified CNF was finally used as a multifunctional crosslinker during the polymerization of NIPAM producing a highly stretchable hydrogel.

**Scheme 13:**
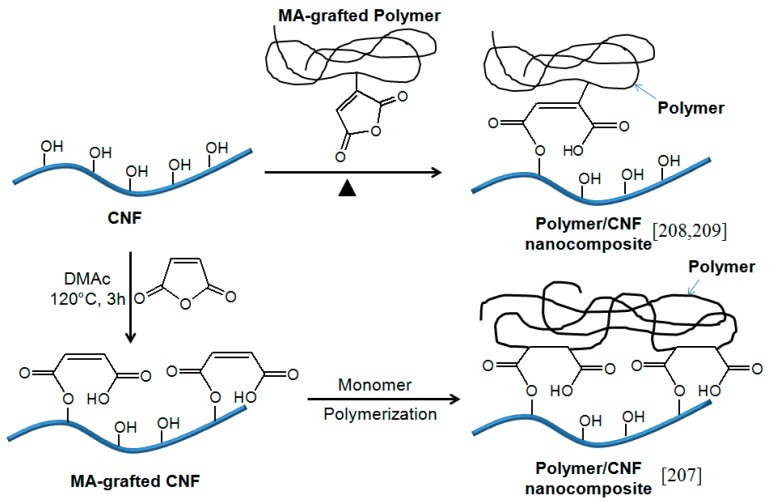
Pathway of esterification reactions for the preparation of well-defined polymer/CNF nanocomposites.

A similar interaction was also found when CNF was used as a reinforcing nanofiller in polystyrene-based block copolymer (BCP), named styrene-ethylene/butylenes-styrene (SEBS) [208]. In this case, the BCP contained 2 wt % attached MA units that took part in the esterification reaction with the CNF hydroxyl groups during the processing at 150 °C (Scheme 13). As a result, in terms of mechanical properties, there was a 35% and 39% increase in tensile strength and the Young’s modulus, respectively, at 2 wt % CNF content. According to Kiziltas and co-workers, there exists ester linkages between the polymer and CNF in poly(styrene-*co*-maleic anhydride) [P(St-*co*-MA)]/CNF nanocomposite [209]. The esterification reaction between surface –OH groups of CNF and the MA-segments of copolymer was expected to happen during the preparation of the nanocomposite by melt compounding at 240 °C.

#### 4.1.6. Surface-Initiated Polymerization

It is known that CAN is a very useful redox initiator for the surface-initiated graft polymerization of acrylates/methacrylates from polysaccharides in aqueous solution [210]. The efficiency of the process is so remarkable that researchers still take the opportunity to develop cellulosic graft copolymers via FRP in water. Littunen and co-workers performed the CAN-initiated polymerization of a series of acrylic monomers, like glycidyl methacrylate (GMA), ethyl acrylate (EA), MMA, BA, and 2-hydroxyethyl methacrylate (HEMA), in the aqueous suspension of CNF, producing graft copolymers of CNF [211]. Grafting efficiency of >80% was observed for GMA and BA, whereas HEMA showed the least. In addition, the graft polymerization of BA produced the longest polymer chain. Ultimately, the approach provided a simple route to fabricate CNF-reinforced polymer nanocomposites where CNF was found to remain completely embedded in the synthetic polymer matrix. 

Apart from CAN, KPS is also believed to initiate the free-radical polymerization from the surface of cellulose through the proton transfer reaction between the sulfate anion radicals and hydroxyl groups. Mahfoudhi and Boufi adopted this process to perform the copolymerization of acrylic acid (AA) and acrylamide (AAm) in the presence of TOCN to produce poly(AA-*co*-AAm)/TOCN nanocomposite hydrogel with very good mechanical properties [212]. 

The surface-initiated ATRP (SI-ATRP) process has acquired significant interest to grow polymer chains from the surface of the nanomaterials. CNF also profoundly offers the scope for the required functionalization to perform SI-ATRP [213]. Wang et al. introduced the –Br functionality to the electrospun regenerated cellulose nanofiber and performed the ATRP of HEMA and sodium acrylate (AAS) to obtain the CNF-based graft copolymer [214]. The membranes obtained from those graft copolymers were successfully applied for the highly-efficient ultrafiltration of nanoparticles (~40 nm) from water. 

**Scheme 14:**
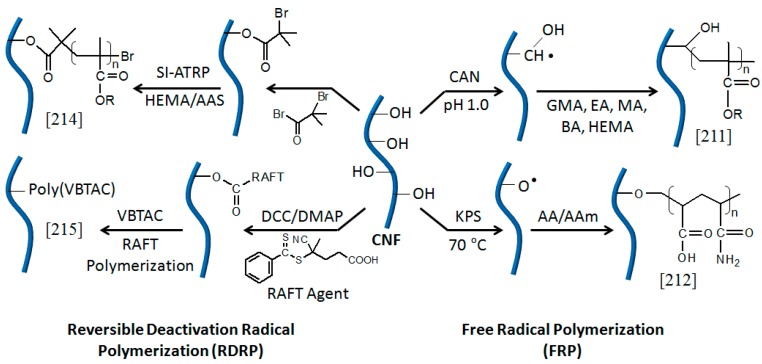
Surface-initiated polymerization approaches to obtain polymer/CNF nanocomposites.

RAFT polymerization was also found to be an important tool for the graft polymerization on CNF. In this case, the RAFT agent 4-cyanopentanoic acid dithiobenzoate (CPAD), having –COOH groups, was first attached with CNF via DCC/DMAP coupling (esterfication with dicyclohexylcarbodiimide (DCC) as a coupling reagent and 4-dimethylaminopyridine (DMAP) as a catalyst) [215]. Afterwards, the polymerization of vinylbenzyl trimethylammonium chloride (VBTAC) in the presence of RAFT-modified CNF produced the cationic graft copolymer which found application in DNA adsorption. Scheme 14 summarizes the different approaches under the surface-initiated FRP and RDRP for the fabrication of well-defined polymer/CNF nanocomposites.

In addition to the surface-initiated radical polymerizations, the surface –OH and –COOH groups of TOCN were successfully utilized for ROP of ɛ-caprolactone (CL) producing PCL-grafted CNF [216].

### 4.2. Polymer/CNF Nanocomposite with Non-Covalent Interactions

#### 4.2.1. Electrostatic Interaction

**Figure 3:**
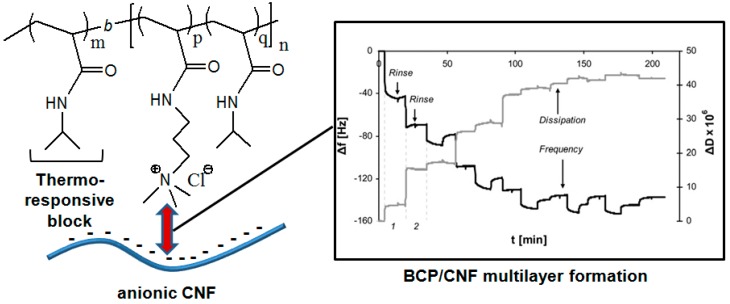
Electrostatic interaction between cationic BCP and anionic CNF and the corresponding QCM–D results for the LbL deposition to produce a BCP/CNF multilayer composite (reproduced with the permission from [218]).

It is very much feasible to fabricate well-defined polymer nanocomposites by virtue of electrostatic interaction between the polymer and nanomaterial. Similar to CNC, CNF also offers the scope to establish such interactions with ionic polymers. However, it is necessary to incorporate the ionic functionality in the CNF surface by chemical pre-treatment, where the popular methods are TEMPO-oxidation, producing TOCN [62] and carboxymethylation [217]. Due to the incorporation of –COOH functionalities CNF became anionic and thereby provided the scope to prepare the nanocomposite of cationic polymer through sufficient electrostatic interaction. In this regard, Utsel and co-workers synthesized a cationic block copolymer and fabricated polyelectrolyte multilayers (PEM) on carboxymethylated-CNF through layer-by-layer (LbL) deposition [218]. The block copolymer was composed of PNIPAM and poly(3-acrylamidopropyl)trimethylammonium chloride (APTAC) where the PNIPAM block was responsible to make the nanocomposite thermo-responsive and the cationic PAPTAC segment provided the required electrostatic interaction with anionic CNF. The adsorption of such cationic polymers to the anionic CNF was analyzed using a quartz crystal microbalance with dissipation monitoring (QCM-D). Figure 3 shows the plausible interaction between the cationic BCP and anionic CNF, resulting in the formation of multilayers, as recorded in the QCM-D plot. The plot represents the adsorption cycle during the LbL deposition. At the initial stage, a large decrease in the frequency was observed, suggesting the high adsorption of BCP onto CNF. Afterwards, the adsorption became slower as it accompanied a certain fraction of desorption. 

Larsson et al. also fabricated a thermo-responsive nanocomposite using the block copolymer composed of quaternized poly(2-(dimethylamino) ethyl methacrylate) (qPDMAEMA) and poly(di(ethylene glycol) methyl ether methacrylate) (PDEGMA) and TOCN [219]. In the nanocomposite, the block copolymer was adsorbed to the TOCN through the cationic PDMAEMA block, where the PDEGMA segment exhibited thermo-responsiveness. Such a type of thermo-responsive nanocomposite may find application as a membrane where the permeability can be controlled by temperature. Electrostatic interaction was successfully utilized to prepare cationic polyethyleneimine (PEI)/carboxymethylated-CNF nanocomposite deposited on a PLA substrate by the LbL process [220]. The resulting PLA films had significantly improved oxygen and water vapour barrier properties. The presence of electrostatically-adsorbed polymers on CNF was identified by FT-IR and TGA studies. Wei et al. prepared a PNIPAM/TOCN composite hydrogel by in situ radical polymerization [221]. In this case, there was substantial electrostatic interaction between the carboxylate of TOCN and the amide of PNIPAM that caused the improved compressive strength and modulus in the composite hydrogel with the increase in the carboxylate content in the TOCN.

#### 4.2.2. Hydrogen-Bonding Interaction

**Scheme 15:**
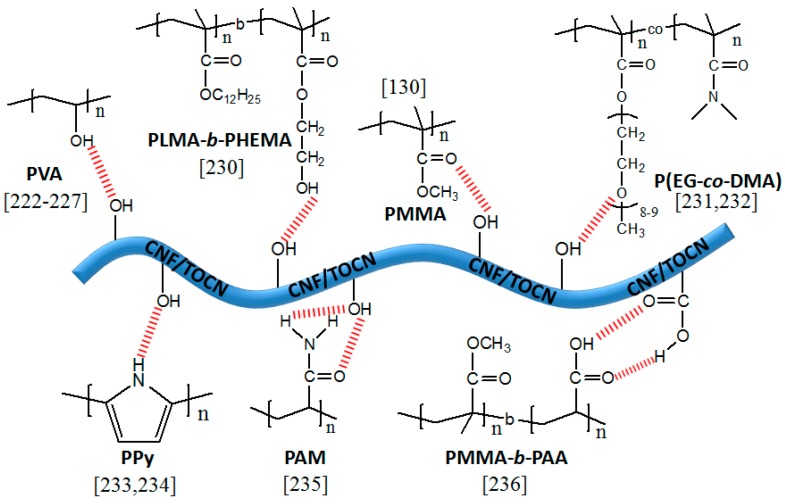
Examples of polymers having a hydrogen bonding interaction with CNF/TOCN to produce the nanocomposites with improved properties.

The presence of numerous –OH groups make CNFs a suitable nanomaterial to be hydrogen-bonded to the polymer producing high-performance nanocomposites. Scheme 15 shows the examples of polymers reported to have H-bonding interaction with CNF in the nanocomposite. Discussing those polymers, first of all, we can mention PVA to have strong H-bonding interaction with cellulosic nanomaterials [222]. Endo and co-workers prepared PVA/TOCN composite fibers by a spinning, drawing, and drying process using an aqueous mixture of PVA and TOCN [223]. Only at 1 wt % TOCN content did the composite drawn fibers exhibit maximum tensile modulus, up to 57 GPa, much higher than the pristine PVA drawn fibers. Due to the formation of H-bonding with amorphous PVA molecules, TOCN was found to be individually dispersed in the PVA matrix without any significant aggregation. The PVA/TOCN composite was also used as an adhesive which exhibited high shear strength, especially at elevated temperature (70 °C) [224]. Cai et al. used cellulose acetate nanofibers to prepare the nanocomposite of PVA [225]. The prepared composite film exhibited improved mechanical properties along with very high visible light transmittance even at high nanofiber loading. The PVA/CNF composite was also used in the development of PE-based composites where PVA acted as a compatibilizer allowing better dispersion of CNF in the PE matrix [226]. In order to obtain a PVA-based nanocomposite with antimicrobial properties, Choo et al. introduced chitosan (CS) in the PVA and dispersed TOCN in that PVA-CS blend matrix [227]. The produced nanocomposite film had improved tensile strength and thermal stability. 

A transparent nanocomposite of PMMA/CNF was prepared by a solution casting process using acetone as the solvent [130]. However, the transparency was reduced with the increase in the CNF loading from 0.25 wt % to 0.5 wt %. In addition, there was no significant change in the thermal stability of the nanocomposite in comparison with pristine PMMA, only an increase in the maximum degradation temperature by 20 °C at 0.25 wt % CNF loading. It raised a concern with the viability of H-bonding interaction between –OH groups of CNF and the carbonyl groups of PMMA.

According to Saba et al., there exists a polymer–filler H-bonding interaction in EP/CNF nanocomposite [228,229]. The nanocomposite exhibited improved thermal and mechanical stability at only 0.75 wt % CNF content. 

Adsorption of poly(lauryl methacrylate)-*b*-poly(2-hydroxyethyl methacrylate) (PLMA-*b*-PHEMA) to CNF made it a suitable nanofiller for HDPE [230]. In this case, the PHEMA segment was believed to have H-bonding interaction with CNF, while the PLMA block was responsible for achieving good dispersion of CNF in the HDPE matrix. The resulting HDPE/CNF nanocomposite had a 140% higher Young’s modulus and an 80% higher tensile strength than neat HDPE. 

CNF/polymer nanopaper was developed by dispersing CNF in the non-ionic copolymer poly[(ethylene glycol methyl ether methacrylate)-*co*-*N*,*N*-dimethylacrylamide] P(EG-*co*-DMA) matrix [231,232]. The nanocomposite was prepared by mixing the CNF into the aqueous solution of the copolymer followed by the evaporation of the water. Finally, the composite nanopaper was obtained, which had tight cohesion due to the H-bonding between PEG segments and CNF, reflected in the 500% increase in elongation at break compared to pristine CNF. 

Lay et al. fabricated polypyrrole (PPy)/CNF composite nanopaper by in situ chemical polymerization using FeCl_3_ as the oxidising agent [233]. In this case, PPy remained coated on the CNF through H-bonding interaction. The produced nanopaper had a very high tensile strength (224 MPa) and elastic modulus (14.5 GPa) in combination with good electrical conductivity (5.2 × 10^−2^ S·cm^−1^). Moreover, the PPy/TOCN composite was successfully used as a hydrophobic and anti-bacterial exploring the inhibitory effect of PPy towards Gram-positive and Gram-negative bacteria [234]. 

Yuan et al. reported the preparation of polyacrylamide (PAM)/CNF hydrogel where the H-bonding interaction between >C=O/N–H parts of PAM and –OH of CNF were found to be the mechanism of reinforcement [235]. The composite hydrogel exhibited elongation at break of 2200% and fracture stress of 1.35 MPa, while, on compression, it can attain up to 99% strain without any breakage. Interestingly, the hydrogel was capable of completely recovering its initial shape immediately after releasing the elongation or compression force. 

**Figure 4:**
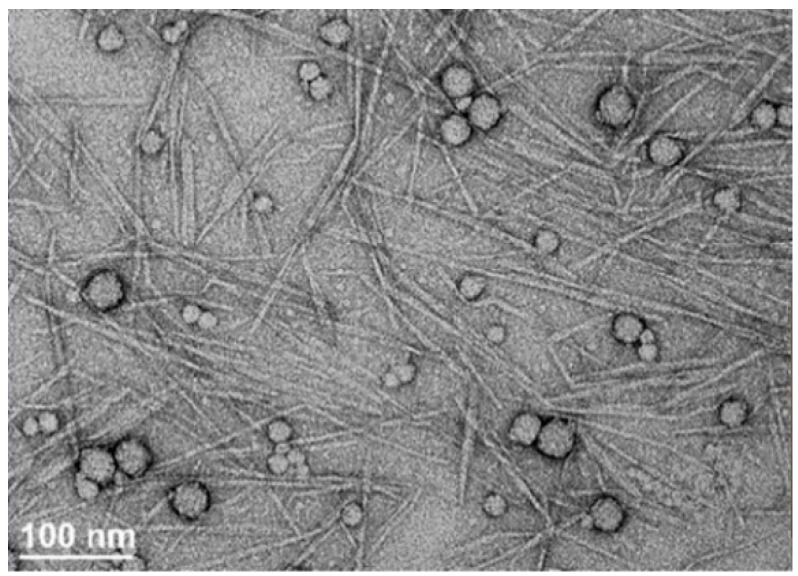
TEM image of the aqueous dispersion of PMMA-*b*-PAA/TOCN composite showing the formation of spherical micelles attached to the nanofibrils (reproduced with the permission from [236]).

The adsorption of an amphiphilic BCP, PMMA-*b*-PAA, onto TOCN was also facilitated by the H-bonding interaction between the –COOH groups of TOCN and PAA [236]. As a result, the aqueous dispersion of PMMA-*b*-PAA/TOCN composite showed the formation of micelles by the amphiphilic BCP and those micelles were found to be attached to the nanofibrils (Figure 4). In addition, a better dispersion of TOCN in organic solvents like DMF, DMSO, ethanol, and methanol was achieved due to the presence of BCP.

#### 4.2.3. Compatibilization through Surface Modification

The highly polar nature of CNF made it difficult to be dispersed in hydrophobic polymer matrices. In such cases where there is no favourable interaction between CNF and polymer, the surface modification of CNF is generally carried out in order to improve the interfacial adhesion between the hydrophilic CNF and hydrophobic polymer [4]. In this section, the authors describe examples of such surface modifications of CNF aiming to achieve polymer–filler compatibility. Table 2 summarizes the type of surface modifier and the respective polymer matrix along with the extent of reinforcement in the nanocomposite. 

Qu et al. performed the surface modification of CNF by 3-methacryloxypropyl trimethoxysilane (MEMO) in ethanol suspension [237]. In this case, the hydrolysed MEMO remained bound to the CNF through H-bonding, as evident from FT-IR analysis. In a similar fashion, the surface modification of CNF was carried out using 3-aminopropyltriethoxysilane (APS) [238]. In both cases, the modified CNF was used as nanofiller for PLA matrix. The improved compatibility between modified CNF and PLA was reflected in the improved mechanical properties of the nanocomposite. APS-modified CNF was also used for the reinforcement of waterborne acrylic resin/polyester blend coating [239]. 

Partially-acetylated CNF was also found to reinforce PLA, especially for food packaging applications [240]. The acetylation reaction was performed using the mixture of acetic acid and nitric acid under boiling conditions for 90 min. The nanocomposite was prepared via solution mixing using DMF as the solvent. The PLA/CNF nanocomposite exhibited substantial improvement in oxygen and water-vapour barrier properties only at 1 wt % CNF content. Importantly, the nanocomposite retained a similar transparency to that of pristine PLA. The flame retardant property of PLA was improved in the nanocomposite with CNF that was modified by the phosphorous-nitrogen based polymer [241]. The authors performed the surface modification of CNF through a multi-step process that consisted of the consecutive reactions with epoxy chloropropane, polyethylenimine, and diethyl phosphate. The obtained PLA/CNF nanocomposite not only had excellent flame-retardancy, but also gained a 24% increase in tensile strength. The surface modification of TOCN by cis-9-octadecenylamine (OA) was performed by Soman and co-workers for the reinforcement of PLA [242]. In this case, the authors carried out the peptidic coupling reaction between TOCN and OA by EDC/NHS. Such a modification of CNF by long alkyl chains aided the compatibility with PLA, as evidenced by the improved thermal and mechanical properties in the nanocomposite.

The surface modification by hexadecenyl succinic anhydride made CNF suitable reinforcing nanofiller for HDPE [243]. The modification reaction was carried out using DMAP/K_2_CO_3_ in NMP solvent, resulting in a DS of up to 0.83. However, the uniform dispersion of CNF was observed for a DS of 0.44, while it worsened at a DS of 0.77. About a 100% increase in tensile strength was achieved in the nanocomposite with respect to the pure HDPE. An ionic liquid with alkyl chains was used by Croitoru and Patachia for the surface modification of CNF and, subsequently, to reinforce HDPE by the modified CNF [244]. In this case, the treatment of CNF by the ionic liquid, named 1-Hexyl-3-methylimidazolium tetrafluoroborate (HMIMBF_4_), was performed by stirring the mixture at 25 °C and 35 °C for 24 h. The HDPE/CNF nanocomposite was obtained by melt compounding at 230 °C. The surface treatment of CNF by the block copolymers, such as poly(dicyclo pentenyloxyethyl methacrylate)-block-poly(2-hydroxyethyl methacrylate) (PDCPMA-*b*-PHEMA) and (PLMA-*b*-PHEMA), was found to be very much successful for the reinforcement of HDPE [230,245]. Here, the PHEMA block had the interaction with CNF, whereas another block was responsible to improve the interfacial adhesion between CNF and HDPE. The nanocomposites had quite an improvement in mechanical properties compared to the pristine HDPE.

**Figure 5:**
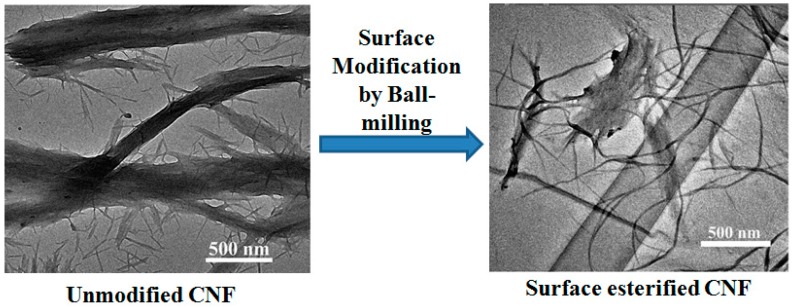
TEM images of unmodified CNF and surface modified CNF, respectively, to show the morphological differences arising due to the surface modification by ball milling (reproduced with the permission from [246]).

Huang et al. adopted the ball milling technique for the surface modification of CNF by *n*-dodecyl succinic anhydride [246]. The modification reaction catalysed by DMAP was carried out in a 40 mL zirconia pot containing seven 10 mm zirconia balls at 200 rpm at room temperature for 2 h to 40 h. Figure 5 shows the TEM images of the unmodified CNF and surface modified CNF dispersed in DMSO. The improved dispersion of nanofibers could clearly be observed upon the surface esterification using the ball milling technique. The modified CNF was used as the reinforcing nanofiller for the PE matrix. The resulting PE/CNF nanocomposite showed a 28% increase in tensile strength compared to PE only. 

The surface treatment of CNF by MMA monomer was found to be beneficial for the development of a PMMA/CNF nanocomposite [247]. The surface treatment was carried out by stirring the mixture of MMA and CNF at 50 °C for 2 h. As a result, the MMA-treated CNF had improved dispersion in an in situ-polymerized PMMA matrix. The nanocomposite had a 15% and 39% increase in tensile strength and Young’s modulus in addition to the improved thermal and moisture resistance properties.

The surface modification of CNF by MA-grafted PP (MAPP) was also found to be beneficial for the reinforcement of PP [248]. In this case, the MAPP emulsion was first mixed with the CNF suspension for 2 min at 2000 rpm, followed by ultrasonic treatment at 80 °C for 1 h. Finally, the emulsion-treated CNF suspension was dried using a spray drying process. The composite of PP and MAPP-modified CNF was prepared using a twin-screw co-rotating extruder. However, there was only an 11% increase in the tensile strength of the nanocomposite compared to the PP. Performing a similar approach, Wang et al. reported a ca. 26% increase in Young’s modulus in the PP/CNF nanocomposite containing 10 wt % CNF and 2 wt % MAPP [249]. Ferrer and co-workers used lignocellulosic fibers for the reinforcement of PP [250]. In this case, the CNF contained about 9% lignin that acted as the compatibilizer resulting in improved dispersion of CNF in the PP matrix. Although, the nanocomposite exhibited only 15% increase in the tensile modulus with respect to the pristine polymer. 

In addition to the chemical aspect, there is a good example of promoting compatibilizing by processing. Iwamoto et al. added 5% MAPP to improve the interfacial adhesion to reinforce PP with lignocellulose nanofibers (LCNF) [251]. The nanofiber used was prepared by the wet-disk milling of wood flour and has not been actively delignified. Solid-state shear grinding (SSSP) using a batch kneader was carried out at a temperature below the PP melting point to improve the dispersion of LCNF in the PP matrix containing 5 wt % maleic anhydride-grafted PP (PP/MAPP/LCNFs = 90:5:5). The nanocomposite was prepared by both compression and injection moulding at 170 °C. There were 9% and 10% increases in the Young’s modulus and yield stress, respectively, in the presence of 5 wt % CNF. It is noteworthy, however, that the tensile strain of the composite after 60-min SSSP reaches 580%, comparable to that of net PP (~750%). In the PP systems reinforced with CNF filler, there are probably no other examples that can maintain such a tensile strain so far. This research example shows that not only is the design of the composite based on the chemical structure, but also the kneading process, important for manifesting the assumed interaction and compatibilizing effect.

#### 4.2.4. Pickering Emulsion

Due to the large specific surface area and high aspect ratio, CNF has the potential to act as a Pickering stabilizer. The inherent hydrophilic nature of CNF made it a suitable Pickering stabilizer for O/W emulsion [252]. A certain degree of surface modification leads to a reduced hydrophilic nature in CNF, which was used for the stabilization of W/O emulsion [253]. The modifiers may include octadecylamine, poly(styrene-*co*-maleic anhydride) (SMA), lauroyl chloride, etc. In this part, we shall discuss the CNF-based polymer nanocomposites prepared via the Pickering emulsion polymerization technique.

**Figure 6:**
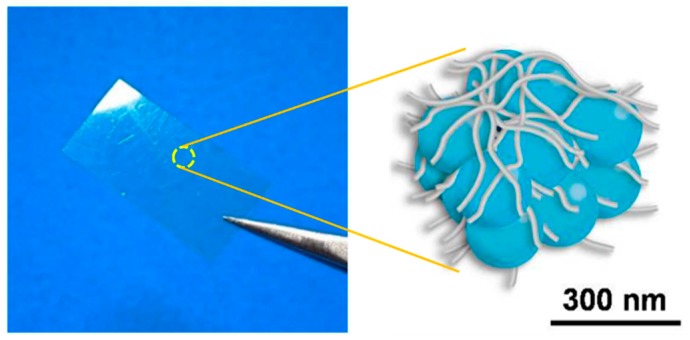
Highly-transparent PS/TOCN nanocomposite film prepared by TOCN-stabilized Pickering emulsion polymerization of styrene (reproduced with the permission from [254]).

Fujisawa et al. recently reported on the PS/TOCN composite prepared via the polymerization of styrene in TOCN-stabilized styrene-in-water emulsion [254]. The emulsion was stable without any creaming due to the repulsive forces between carboxyl groups of TOCN. The obtained PS microspheres covered by nanofibrils were introduced by hot-pressing at 160 °C and 3 MPa, producing a transparent PS/TOCN composite (Figure 6). In this case, the content of TOCN was 12 wt % of the PS. Although there was no evidence for any sort of covalent bonding or non-covalent interaction between the TOCN and PS, the Pickering stabilization approach led to the homogeneous dispersion of TOCN in the PS matrix.

In another example, the combination of unmodified and surface-modified CNF was successfully used for the simultaneous stabilization of O/W and W/O emulsions, producing a W/O/W emulsion system [255,256]. In this case, styrene-in-water emulsion was stabilized by the unmodified CNF and the CNF modified by SMA copolymer stabilized water droplets inside the styrene droplets. Interestingly, the surface modification of CNF by SMA happened in situ, i.e., during the polymerization. Due to the partial hydrophobic nature, the modified CNF became encapsulated inside the styrene droplet and stabilized the water droplets. After the polymerization, the aqueous dispersion of PS microspheres was obtained where the PS microspheres also contained water droplets inside. This approach guided the development of water-expandable PS/CNF nanocomposites from which low-density PS foam could be produced.

The Pickering stabilization approach was also successfully utilized in the fabrication of a PLA/CNF nanocomposite [257]. In this case, PLA dissolved in dichloromethane was emulsified by CNF and the nanocomposite was obtained after complete evaporation of the solvent upon keeping the emulsion at ambient temperature for 24 h. The nanocomposite had quite an improvement in thermal and mechanical properties compared to pristine PLA. 

## 5. Summary and Outlook

This review describes the recent reports on polymer/nanocellulose composites through classifying them under specific modes of interaction between the polymer and the nanocellulose. The only trouble in fabricating polymer/nanocellulose composites is the polar nature of nanocellulose that makes them difficult to disperse in hydrophobic polymer matrices. Therefore, it is necessary to incorporate any sort of interaction between the polymer and the nanocellulose. This review is a comprehensive guide to understand the selection criteria for the polymeric component and the process to attain the desired polymer-nanocellulose interaction, producing the nanocomposite. In order to achieve a covalent interaction between the polymer and nanocellulose, the surface-initiated FRP, RDRP, and ROP were found to be the most efficient approaches, while hydrogen bonding interaction was very much effective under non-covalent interaction. Introducing ‘click reaction’ and UPy functionalities are appealing approaches to incorporate the self-healing nature to the nanocomposites. There were also several examples to improve the polymer-nanocellulose compatibility through the specific surface modification of nanocellulose. Due to the relatively small aspect ratio and ease of dispersibility, CNC was more commonly used than CNF. However, the Pickering stabilization using CNF was found as a promising approach. 

As cellulose is almost inexhaustible as a resource, the development of cellulosic nanomaterials and their application for the reinforcement of a synthetic polymer matrix are not only beneficial from a commercial viewpoint, but also to the environment. From the number of articles that have been taken into account, we can see the increasing trend in the research on polymer/nanocellulose composites over the past few years. In the upcoming years, the research will not be limited to academic publications, as we can see glimpses of the future prospects of nanocellulose through industrial production [21]. This review will be of prime interest to polymer and material scientists looking for an introduction to nanocellulose for the preparation of polymer nanocomposites. If robust polymer science is being developed, nanocellulose research will be developed. Even in countries and regions where nanocellulose research is not yet active, it is expected that the research scopes will become active mainly with polymer scientists.

In terms of the commercial aspects, cellulosic nanomaterials are still expensive due to the high cost of production. In addition, high moisture content inevitably accompanying the production of nanocellulose is a problem. It is undeniable that the solvent substitution step also leads to an increase in cost. The supply system of nanocellulose is being established in countries where its research is precedent. By analogy with general theory, cost reductions can be expected by large-scale production. In order to reach large-scale production, exploitation of application is important. For that, we need to pursue what we cannot do without nanocellulose. In addition to designing attractive composites in terms of functionality and performance, further brush-ups are required to streamline the process of compounding. The authors hope that nanocellulose production and use will progress widely not only in countries promoting its research and development, but also in developing countries with abundant cellulose resources. If this review contributes to that, it is an unexpected pleasure.

## Figures and Tables

**Table 1:** Summarizing the type of surface modifier for CNC and the corresponding polymer matrix to prepare the nanocomposite, also including the extent of reinforcement in the nanocomposite.

Surface Modifier for CNC	Compatible Polymer	Key Improvements in the Nanocomposite	Ref.
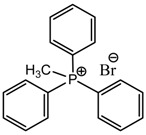 methyl(triphenyl) phosphonium bromide (MePh_3_PBr)	Epoxy/PS	No improvement in mechanical properties. No change in *T*_g_.	Fox et al. [168]
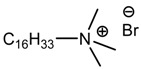 hexadecyltrimethyl ammonium bromide (QS)	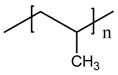 PP	Up to 54% increase in Young’s modulus. Up to eight-fold increase in elongation at break.	Nagalakshmaiah et al. [170]
–do–	UV-curable acrylic coating	Decrease in WVTR. Increase in optical clarity.	Kaboorani et al. [171]
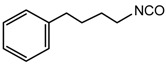 phenylbutyl isocyanate	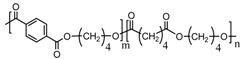 poly(butylene adipate-*co*-terephthalate) (PBAT)	55% increase in elastic modulus. 63% decrease in water vapour permeability.	Morelli et al. [180]
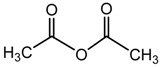 acetic anhydride	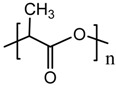 PLA	Increase in tensile strength and elongation at break.	Myoung et al. [188]
–do–	Amine-cured epoxy resin	73% increase in tensile strength. 98% increase in modulus.	Abraham et al. [146]
–do–	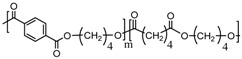 PBAT	Three-fold increase in the Young’s modulus. No change in tensile strength and decrease in % elongation at break.	Zhang et al. [189]
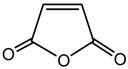 maleic anhydride (MA)	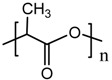 PLA	Increased hygroscopic property.	Parize et al. [190]
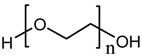 polyethylene glycol (PEG)	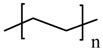 HDPE	Improved mechanical properties.	Inai et al. [172]
–do–	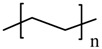 LDPE	Improved thermal stability.	Azouz et al. [173]
–do–	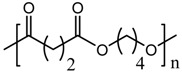 Polybutylene succinate (PBS)	Slight increase in elongation at break.	Ludueña et al. [177]
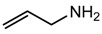 allyl amine (Reaction with COOH–CNC)	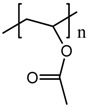 Polyvinyl acetate (PVAc)	500% increase in wet tensile modulus after UV-crosslinking.	Fox et al. [192]
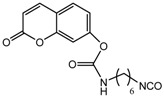 NCO-terminated Coumarin	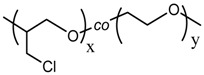 Poly(epichlorohydrin-*co*-ethylene oxide)	50-fold increase in storage modulus at 25 °C.	Biyani et al. [181]
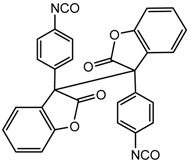 NCO-terminated diarylbibenzofuranone (DABBF)	DABBF-based crosslinked polymer	Mechanochromic and self-healing property.	Imato et al. [184]
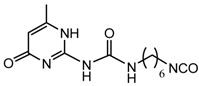 NCO-terminated UPy-	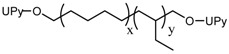 UPy-functionalized poly(ethylene-*co*-butylene)	Up to 12-fold increase in the Young’s modulus.	Biyani et al. [182]
–do–	LDPE LLDPE SBS EO-EPI	Improved stiffness and strength.	Natte-rodt et al. [183]
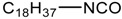 *n*-octadecyl isocyanate	PLA/NR blend	Decrease in Young’s modulus and tensile strength.	Bitinis et al. [179]
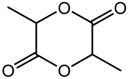 L-Lactide	–do–	No change in Young’s modulus and tensile strength.	–do–
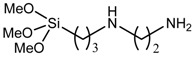 *N*-(β-aminoethyl)-ɣ-aminopropyltrimethoxysilane (APTES)	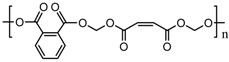 Unsaturated polyester resin (UPR)	96% increase in toughness.	Kargarz-adeh et al. [185]
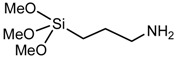 (3-aminopropyl) trimethoxysilane	Epoxy resin	Up to 7.7-fold increase in storage modulus at 160 °C.	Yue et al. [187]
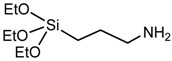 (3-aminopropyl) triethoxysilane	Polyimide	40% increase in tensile modulus.	Lee et al. [186]

**Table 2:** Summarizing the type of surface modifier for CNF and the corresponding polymer matrix to prepare the nanocomposite, also including the extent of reinforcement in the nanocomposite.

Surface Modifier for CNF	Compatible Polymer	Key Improvements in the Nanocomposite	Reference
CH_3_COOH acetic acid	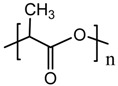 polylactic acid (PLA)	10% increase in elongation. 64% and 46% decrease in oxygen and water-vapour transmission rate.	Trifol et al. [240]
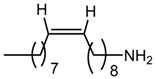 cis-9-octadecenylamine (OA)	–do–	Improved thermal and mechanical properties.	Soman et al. [242]
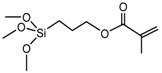 3-methacryloxypropyl trimethoxysilane (MEMO)	–do–	42.3% increase in tensile strength. 28.2% increase in elongation.	Qu et al. [237]
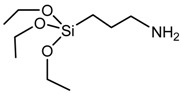 3-aminopropyl triethoxysilane (APS)	–do–	11.8% increase in modulus. 34.5% increase in hardness.	Frone et al. [238]
–do–	Waterborne acrylic resin/polyester blend	Retained high transparency ~90%. 500% increase in the Young’s modulus.	Tan et al. [239]
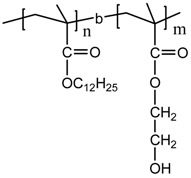 PLMA-*b*-PHEMA	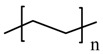 HDPE	84.0% increase in tensile strength. 140% increase in the Young’s modulus.	Sakakibara et al. [230]
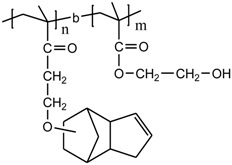 PDCPMA-*b*-PHEMA	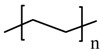 HDPE	420% increase in the Young’s modulus. 200% increase in tensile strength.	Sakakibara et al. [245]
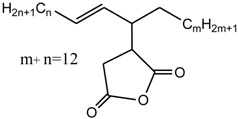 alkenylsuccinic anhydride	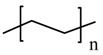 HDPE	100% increase in tensile strength. 86% increase in modulus.	Sato et al. [243]
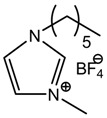 1-Hexyl-3-methyl imidazolium tetrafluoroborate (HMIMBF_4_)	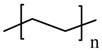 HDPE	–	Croitoru et al. [244]
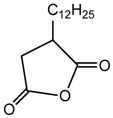 n-dodecylsuccinic anhydride	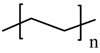 PE	28% increase in tensile strength. 83% increase in the Young’s modulus.	Huang et al. [246]
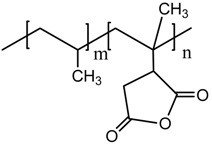 maleic anhydride-grafted polypropylene (MAPP)	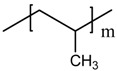 PP	11% increase in tensile strength. 23% increase in impact strength.	Peng et al. [248]
–do–	–do–	5.9% increase in flexural strength. 26% increase in the Young’s modulus.	Wang et al. [249]
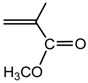 methyl methacrylate (MMA)	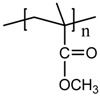 PMMA	15% increase in tensile strength. 39% increase in the Young’s modulus.	Banerjee et al. [247]
